# The involvement of type IV pili and the phytochrome CphA in gliding motility, lateral motility and photophobotaxis of the cyanobacterium *Phormidium lacuna*

**DOI:** 10.1371/journal.pone.0249509

**Published:** 2022-01-27

**Authors:** Tilman Lamparter, Jennifer Babian, Katrin Fröhlich, Marion Mielke, Nora Weber, Nadja Wunsch, Finn Zais, Kevin Schulz, Vera Aschmann, Nina Spohrer, Norbert Krauß

**Affiliations:** Karlsruhe Institute of Technology KIT, Botanical Institute, Karlsruhe, Germany; Texas A&M University, UNITED STATES

## Abstract

*Phormidium lacuna* is a naturally competent, filamentous cyanobacterium that belongs to the order Oscillatoriales. The filaments are motile on agar and other surfaces and display rapid lateral movements in liquid culture. Furthermore, they exhibit a photophobotactic response, a phototactic response towards light that is projected vertically onto the area covered by the culture. However, the molecular mechanisms underlying these phenomena are unclear. We performed the first molecular studies on the motility of an Oscillatoriales member. We generated mutants in which a kanamycin resistance cassette (KanR) was integrated in the phytochrome gene *cphA* and in various genes of the type IV pilin apparatus. *pilM*, *pilN*, *pilQ* and *pilT* mutants were defective in gliding motility, lateral movements and photophobotaxis, indicating that type IV pili are involved in all three kinds of motility. *pilB* mutants were only partially blocked in terms of their responses. *pilB* is the proposed ATPase for expelling of the filament in type IV pili. The genome reveals proteins sharing weak *pilB* homology in the ATPase region, these might explain the incomplete phenotype. The *cphA* mutant revealed a significantly reduced photophobotactic response towards red light. Therefore, our results imply that CphA acts as one of several photophobotaxis photoreceptors or that it could modulate the photophobotaxis response.

## Introduction

Around 3.0 billion years ago, cyanobacteria evolved the ability to perform oxygenic photosynthesis [1]. This invention caused a rise in atmospheric oxygen that changed the diversity and complexity of species on earth dramatically. The plastids of all eukaryotic photosynthetic organisms are descendants of a cyanobacterial endosymbiont [2, 3].

Four kinds of cellular organizations are recognized in cyanobacteria, namely single celled species, linear filaments, linear filaments with heterocysts and branched filaments. Single celled and filamentous cyanobacteria can move across surfaces using a gliding mechanism [4–6]. For members of the Nostocales [7], i.e. filamentous cyanobacteria with heterocysts, this motility is often restricted to hormogonia [4, 8], a cell type formed during environmental stress or in symbioses with plants [9]. Most genetic and molecular studies on cyanobacterial motility have been performed with the single celled species *Synechocystis* sp. PCC 6803 and *Synechococcus elongatus* PCC 7924 [10–12] and one member of Nostocales [7], *Nostoc punctiforme* [8, 13]. Type IV pili act as driving complexes for gliding motility (often referred to as twitching motility) and have been intensely investigated in other bacteria such as *Pseudomonas aeruginosa* [14] or *Neisseria meningitidis* [15]. The type IV pilus driven movement is based on a retraction and extrusion of protein filaments. The PilA protein, formed from the PilA precursor protein *via* cleavage by the protease PilD, concatenates to build extracellular pili that are expelled or retracted by motive force generated inside the cell. The ATPases PilB and PilT function as motor proteins for expelling and retraction, respectively, and PilQ forms channels that facilitate PilA translocation through the outer membrane [16, 17]. PilC, PilM and PilN are involved in the assembly process at the inner membrane [16, 18]. Depending on the organism, several other Pil proteins may contribute to channel formation or assembly [18].

Cyanobacteria can express several PilA homologs. In *Synechocystis* sp. PCC 6803, PilA1 is required for motility, as gene knockouts showed a nonmotile phenotype. *Synechocystis* sp. PCC 6803 expresses nine PilA homologs, which are termed minor pilins and serve different roles. Inactivation of some of these also led to non-motile cells [19, 20]. Knockouts of *pilC*, *pilD* and *pilT1* also had a nonmotile phenotype, whereas *pilA2* and *pilT2* knockouts were still motile [10]. For *Nostoc punctiforme* it was found that motility is lost in mutants in which *pilB*, *pilN* or *pilQ* were defective [8]. Type IV pili are also regarded as important machinery for DNA uptake in natural transformation [18, 21, 22] and in this context, cyanobacterial genomes have recently been screened for pilin genes [21, 23, 24]. Almost all out of 400 analyzed cyanobacteria were found to have a complete set of type IV pili genes. This wide distribution suggests that type IV pili could be the apparatus that drives gliding motility in all cyanobacteria, however, in filamentous species belonging to the Oscillatoriales order [7] the role of type IV pili has as yet not been analyzed and other mechanisms for movement have been proposed [25]. Besides gliding movement and DNA uptake, other functions have been described in cyanobacteria in which type IV pili are involved, such as flocculation [20] or floating in water column [26].

What is the evolutionary background of the gliding movement of cyanobacteria? Many cyanobacteria move either towards the light or away from the light in a process termed phototaxis. This effect maximizes photosynthetic light capture or protects the cyanobacterium from damage of the photosynthetic apparatus if the light is too strong, respectively. Phototaxis requires one or several photoreceptors for light sensing and a mechanism to transform the light direction into directional action. It can be difficult to distinguish between photoreceptor, the first protein in the signal transduction chain, and light sensing modulating proteins. Loss of both kinds of proteins by mutagenesis result in a modified phototaxis. A regulation of phototaxis through photosynthesis has also been postulated for filamentous cyanobacteria [13, 27].

In *Synechocystis* sp. PCC 6803 [28–30] Cph2, PixJ, PixD and UirS have been identified as photoreceptors or as modulating proteins of phototaxis [31, 32]. Cph2, PixJ and UirS are cyanobacteriochromes, which are defined by one or several GAF domains with a bilin chromophore [33]. PixJ has also a methylaccepting chemotaxis domain, like chemosensors in the chemotaxis response. UirS carries a C-terminal histidine kinase [32]. PixD is a flavin-binding BLUF protein. This protein interacts with PixE, a response regulator-like protein, which in turn interacts with PilB and could thereby modulate phototaxis [34]. Knockout of *cph2*, *pixD*, *uirS* or *pixE* results in an induction of phototaxis or reversion of the phototaxis direction at particular wavelengths [32, 35].

In *Synechocystis* sp. PCC6803, a light focusing effect results in a high light intensity on the light avoiding side of the cell [30]. This focusing mechanism has been confirmed for phototaxis of *Synechococcus elongatus* PCC 7924 [12]. In this species, a PixJ homolog with 5 GAF domains is the proposed photoreceptor; phototaxis is lost in the *pixJ* mutant [12].

Regular cyanobacterial phytochromes such as Cph1 of *Synechocystis* sp. PCC 6803 [36], have not been reported to be involved in phototaxis. The difference between phytochromes and cyanobacteriochromes such as PixJ lies in their domain arrangements and their spectral properties. A regular phytochrome consists of PAS, GAF and PHY domains and a C-terminal output module such as a histidine kinase. Cyanobacteriochromes lack the PAS or the PHY domain or both, but carry other domains in high variability. In phytochromes and in cyanobacteriochromes, a bilin chromophore is bound to the GAF domain. This chromophore undergoes light-triggered changes between two stable spectral forms. In phytochromes these are red- and far-red absorbing forms, whereas in cyanobacteriochromes, different spectral forms are realized.

Our group has isolated several strains of *Phormidium lacuna* from marine rockpools, characterized growth and other parameters and sequenced the genomes. *Phormidium lacuna* is a filamentous species without heterocysts that belongs to Oscillatoriales. According to 16S rRNA based phylogenetic studies [24], this species is located in the clade C4 according to [37]. *Phormidium lacuna* is only distantly related to the model species *Synechocystis* sp. PCC 6803 or *Nostoc punctiforme*. Directly after isolation from the wild, it became obvious that *Phormidium lacuna* filaments are motile on agar surfaces, but a phototaxis response was not observed. *Phormidium lacuna* contains one typical phytochrome with PAS, GAF and PHY domains and 16 cyanobacteriochrome-like proteins that have one or more GAF domains with a chromophore binding cysteine [38]. Five of these reveal close partial BLAST homology with PixJ of *Synechocystis* sp. PCC 6803. The closest homolog (WP_087710364.1) has four GAF domains and a C-terminal MCP (methyl-accepting chemotaxis protein) domain, but the others have no MCP domain. No PixD or PixE homologs were found. *Phormidium lacuna* harbors also all genes for type IV pili [24].

Molecular studies on filamentous cyanobacteria are often hampered by the difficulty to perform genetic manipulations. During trials for gene transformation by electroporation, we found that *Phormidium lacuna* cells can take up DNA naturally and integrate it into chromosomes by homologous recombination. This was the first successful natural transformation of an Oscillatoriales member [24]. After this discovery we aimed at disruption of pilin genes and the phytochrome gene in *Phormidium lacuna* by insertional mutagenesis. We made disruptant mutants of *pilA1*, *pilB*, *pilD*, *pilM*, *pilN*, *pilQ* and *pilT*, and the phytochrome gene *cphA*. These mutants were investigated for longitudinal movement on agar and lateral movement in liquid medium. During our studies we found experimental conditions for observing phototaxis towards vertical light, also termed photophobotaxis, so that we could compare the tactic responses of mutants and wild type. Type IV pili are clearly involved in all three kinds of movements of *Phormidium lacuna* and CphA could play a photoreceptor role or modulating role in phototaxis and surface attachment.

## Methods

### Culture of *Phormidium lacuna*

The strain HE10DO of *Phormidium* lacuna was used for all experiments. The genome of the strain HE10JO has been sequenced earlier. The recently sequenced genome of HE10DO differed in only ca. 1000 bases from the published HE10JO sequence. The filaments were cultivated in f/2 seawater medium [38, 39] in 20 ml or 50 ml culture flaks under continuous white LED light of 300 μmol m^-2^ s^-1^ under agitation at 25°C.

### Construction of disruption vectors and transformation

The construction of the *chwA* mutant was described earlier [24]. The gene was previously termed “7_37”. The name ChwA stands for a protein with a “Clostridial hydrophobic with conserved tryptophan (ChW)” domain [40]. For construction of the other mutants, ca. 2000 bp of each coding sequence (plus, when necessary, upstream sequence) were amplified from genomic DNA by PCR using the primers as listed in S1 Table. Each sequence was cloned into the pGEMT easy *E*. *coli* vector (Promega, Madison, WI, USA) and the plasmid linearized by PCR using primers that bind to the center of the cloned sequence (S1 Table). The KanR cassette was cloned into this sequence by sticky end cloning using restriction enzymes that can be recognized in the primer sequences. The positions where the sequences are interrupted 3’ of the start codon are given in Table 1. For transformation of *Phormidium lacuna*, the respective *E*. *coli* vector was purified by midi prep (Macherey Nagel, Düren, Germany). *Phormidium lacuna* was cultivated in 100 ml f/2 medium until A_750 nm_ reached 0.35 (ca. 5 d). The cell culture was centrifuged at 6000 × g for 3 min and the major part of the supernatant was discarded. A small portion of the supernatant was used to bring the volume of the pelleted cells to 800 μl. Ten μg vector DNA were mixed with 100 μl cell suspension and pipetted in the center of an f/2 agar plate (1.5% Bacto agar and 75 μg/ml Kn). This procedure was repeated to get 8 Petri dishes with DNA and *Phormidium cells*. The Petri dishes were sealed with Parafilm. After 2 weeks, filaments were typically transferred to plates with 500 μg/ml kanamycin (Kn). After another 2 weeks, single growing filaments were isolated and cultivated on fresh agar plates with 500 μg/ml Kn. Propagation continued in liquid medium with 100 μg/ml Kn. Kn concentrations and time schedules varied slightly between transformation experiments. To test for integration into the homologous site and completeness of segregation, we used inner and outer primer pairs. The inner primers were identical with the primers used for vector construction and bind at the 5’ and 3’ ends of the integrated sequence, the outer primers bind just 5’ and 3’ outside the integrated sequence, respectively (see S1 Table for primers). When segregation was complete (no more wild type band visible), the mutant was used for physiological assays. Segregation of *pilA1’* was incomplete over the entire observation time of several months and during the motility experiments. Disruption of *pilD* was not possible. Details are given in the Results section. For cultivation, mutants were always grown in medium with 100 μg/ml Kn, for experiments the cells were always transferred into medium without antibiotics (S1 Fig for PCR data).

**Table 1:** *Phormidium lacuna* proteins and genes addressed in the present study. In the third column, the lengths of the protein sequences and the identities with *Synechocystis* sp. PCC 6803 protein homologs [55] are given. The fourth column gives the (proposed) position of the insertion of the KanR resistance cassette and the length of the entire open reading frame (ORF) in the number of base pairs.

Protein name	Proposed function	# amino acids/ identity with Synechocystis	Position of integration / length of ORF in # bp
ChwA	Hypothetical, ChW repeats		531 / 1143
PilA1	Precursor for pili protein	173 / 39%	19 / 513
PilB	ATP ase motor protein, extension	676 / 63%	36 / 2016
PilD	PilA peptidase, inner membrane	271 / 50%	89 / 252
PilM	Pilin pore forming, inner membrane	373 / 55%	27 / 1116
PilN	Pilin pore stabilizing inter membrane	306 / 23%	480 / 783
PilQ	Pore forming outer membrane	909 /35%	1081 / 2532
PilT	ATP ase motor protein, retraction	379 / 69%	21 / 948
CphA	Photoreceptor	764 / 44%	1103 / 2292

### Motility experiments

For all experiments on motility, *Phormidium lacuna* was transferred to Kn-free liquid f/2^+^ medium and cultivated for 5 days until A_750 nm_ reached 0.35–0.4. The PCR pattern of the integration site (S1 Fig) remained unchanged during this non-selective growth. The filaments were then treated with an Ultraturrax rotating knife (Silent Crusher, Heidolph, Schwabach, Germany) at 10000 rpm for 3 min. For motility studies on agar, filaments were usually concentrated 10-fold by centrifugation and 100 μl of the solution were dispersed on a Bacto agar f/2 Petri dish with a diameter of 5 cm. Images were recorded in 1 min intervals using a conventional microscope and a Bressler (Rhede, Germany) ocular “full HD” camera. The intensity of the microscope light was 250 μmol m^-2^ s^-1^. In some experiments, the Ultraturrax treated filaments were directly pipetted onto a 5 cm petri dish with agar medium, kept for 4 h in a growth chamber and photographed through a microscope.

For studying movement in liquid f/2 culture, 8 ml filament suspension (OD _750 nm_ = 0.4) were transferred into a 5 cm Petri dish without agar. The movement of the filaments was observed through a Leica DM750 microscope and recorded with an EC3 camera for 30 s (no time lapse). Of each sample, recordings at 3 to 10 different characteristic positions were taken.

For photophobotaxis experiments, we constructed a plastic holder in which a 5 mm wide, round LED is mounted at one end of a 15 mm long vertical tube with the light beam pointing upward. For blue, green and red light, LEDs with maximum emissions at 470 nm, 520 nm and 680 nm were chosen, respectively. The light intensity was 15 μmol m^-2^ s^-1^ unless indicated otherwise. Eight ml Ultraturrax treated *Phormidium* suspension (OD _750 nm_ = 0.4) were pipetted into a 5 cm Petri dish which was then placed on the LED-holder so that the LED is in the middle of the petri dish. The samples were kept for 1, 2 or 3 d in a dark room. Typically, the LED irradiation results in the formation of a circular area that is covered with circle of filaments in the center of the Petri dish. After LED irradiation, the Petri dish was photographed at its original position, a movement of the Petri dish could partially destroy the circular shape. The Petri dish was then shaken manually for 3 s and photographed again. The diameters of the circles were measured with ImageJ (NCBI). To this end, the image was loaded with ImageJ and a measuring line was drawn through the center of the petri dish. From the profile of the line, the diameter of the central circle was determined by the distance between half-maximal changes of pixel intensities. Sometimes filaments formed aggregates that were not the result of photophobotaxis. These were not considered in our evaluations. If only aggregates were observed and no evidence for photophobotaxis was found, the diameter of sample was recorded as zero. Photophobotaxis based aggregation could be clearly distinguished from other aggregations by the position within the Petri dish and by the fact that only photophobotaxis based aggregation results in surface attachment.

## Results

### Generation of insertion mutants

Type IV pili are involved in gliding motility of many bacteria including single celled cyanobacteria and members of the Nostocales (filamentous species with heterocysts). To see how type IV pili are involved in a member of Oscillatoriales (filamentous species without heterocysts), we aimed at disrupting genes of PilA1, PilB, PilD, PilM, PilN, PilQ and PilT in the genome of *Phormidium lacuna*. The sequences were identified as BLAST homologs of *Synechocystis* sp. PCC 6803 and other bacteria [24]. The degree of homology between *Synechocystis* sp. PCC 6803 and *Phormidium lacuna* proteins is given in Table 1. We also disrupted the phytochrome gene of *Phormidium lacuna* in order to test for a possible role of this photoreceptor in motion. Phytochromes are abundant in most cyanobacteria, but their biological role in these organisms is unclear [41]. *Phormidium lacuna* contains one phytochrome, which is denominated CphA here, in accordance with CphA from *Fremyella diplosiphon* [42]. In the present study, we did not address other possible photoreceptor proteins such as Cph2 [11] or PixJ [12, 43].

As a control for possible effects on the KanR cassette we used a mutant in which the gene of ChwA is disrupted. *chwA*, originally termed 7_37 (open reading frame 37 on scaffold 7), was the first gene of *Phormidium lacuna* for which natural transformation and homologous integration of a KanR cassette was successful [24]. The protein is annotated in the NCBI database as hypothetical protein. It has 3 ChW [40] repeats, hence the name ChwA in the present study.

For gene disruptions, cloning vectors were constructed that carried a sequence of 2000 bases derived from the gene of interest and the region upstream of the start codon. The KanR cassette was placed in the center of this sequence, so that there are 1000 bp of homologous sequence on both sides of the resistance cassette.

In this way, we built disruption vectors for *pilA1*, *pilB*, *pilD*, *pilM*, *pilN*, *pilQ*, *pilT* and *cphA* and obtained resistant transformants for all insertion constructs. For *pilB*, *pilM*, *pilN*, *pilQ*, *pilT* and *cphA* genes, the integration into the expected site and complete segregation into all chromosomes was confirmed by PCR using inner and outer primers that bind to chromosomal sequences inside and outside the integration vector, respectively (see S1 Table for list of primers and S1 Fig for PCR with inner and outer primers). The PCR pattern remained during 7 days on non-selective medium, the maximum time that was used for physiological experiments. In case of *pilA1*, 7 out of 8 resistant strains showed a PCR double band indicative of partial integration, *i*.*e*. one part of chromosomes with interrupted sequences and the other part with wild type *pilA1* (S1 Fig). The incomplete segregation pattern remained unchanged over many months in selective growth medium. Complete segregation of *pilA1* insertion is thus impossible, indicating that the complete loss of *pilA1* would be lethal. Although only limited information can be gained from such mutants, we performed also experiments with one of these strains. This strain is termed *pilA1’*. Another resistant strain had only the wild type *pilA1* band. In this strain, the KanR sequence must have integrated into another site. All these mutants show that disruption of *pilA1* and complete segregation is lethal.

*pilD* knockout trials yielded only two resistant strains out of 4 transformations, although the same transformation effort was undertaken as with the other genes. During the first two weeks of selection, both mutants grew very slowly, but after another 2 weeks, the filaments recovered and grew at normal speed. In these strains, the *pilD* gene was not interrupted. We assume that the slow growth is caused by disruption of *pilD* genes in a certain proportion of the chromosomes and that by a second recombination event, the KanR cassette moved to another site. This suggests that the loss of *pilD* is lethal. The *pilD* knockout of *Synechocystis* sp. PCC 6803 is also lethal, although the cells can survive on glucose [44]. We did not try selection on glucose medium.

### Motility on agar

The motility on agar of *Phormidium lacuna* was usually investigated with filaments that were treated with an Ultraturrax rotating knife. This treatment separates adjacent filaments of the liquid preculture and cuts long filaments into shorter ones. Filaments were then brought onto agar surface and images were recorded in 1 min intervals through a microscope for several h. These microscope observations were made with white light at an intensity of 250 μmol m^-2^ s^-1^.

**Fig 1:**
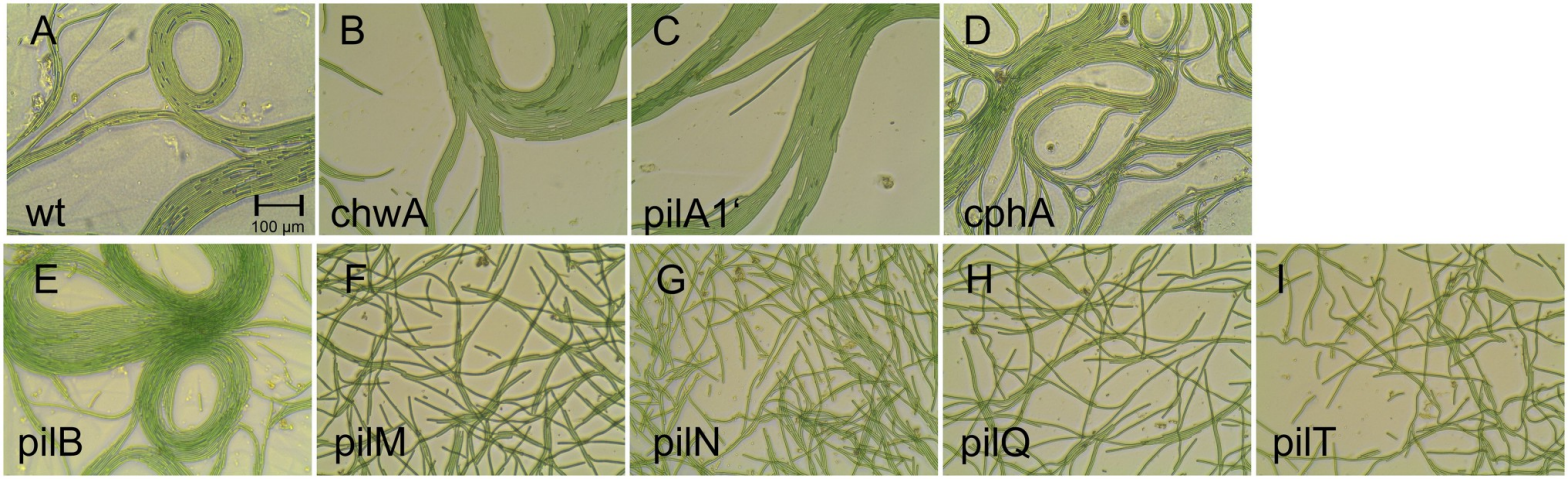
*Phormidium lacuna* filaments were treated with an Ultraturrax and placed for 4 h on agar surface. The mutant names are given in each panel.

After placement on the agar surface, all wild type filaments started to move instantaneously. The direction of movement was always longitudinal towards one end of the filament. The polarity of movement, indicating whether a filament moves towards one or the other tip, switched frequently in an apparently random manner (S1 Movie). The movement of the filaments was not completely straight but in circle segments with variable diameter. We often observed filaments that described a complete circle on which they moved for several rounds. Each filament left a visible trace of extruded material on the agar surface (examples can be seen in Fig 1A–1E in the areas that are not covered by filaments) which could serve as guide for other filaments or the same filament when it returned to the same position. When a filament touched another filament, it aligned its longitudinal direction so that finally long parts of both filaments were parallel and adjacent. Generally, filaments remained together, and additional filaments joined so that bundles of many filaments were formed (S1 Movie and Fig 1A–1E). These filament bundles were usually one cell layer thick, sometimes a second layer of few filaments located above the lower one. More than two layers were not observed. The parallel leaf like filament arrangement of the bundles was highly flexible. All filaments moved continuously, also versus each other, bundles joined and separated, and higher ordered structures like circles were formed. Clearly, bundle formation and bundle dynamics are important functions of filament movement and communication between the filaments is required for such coordinated movements.

**Fig 2:**
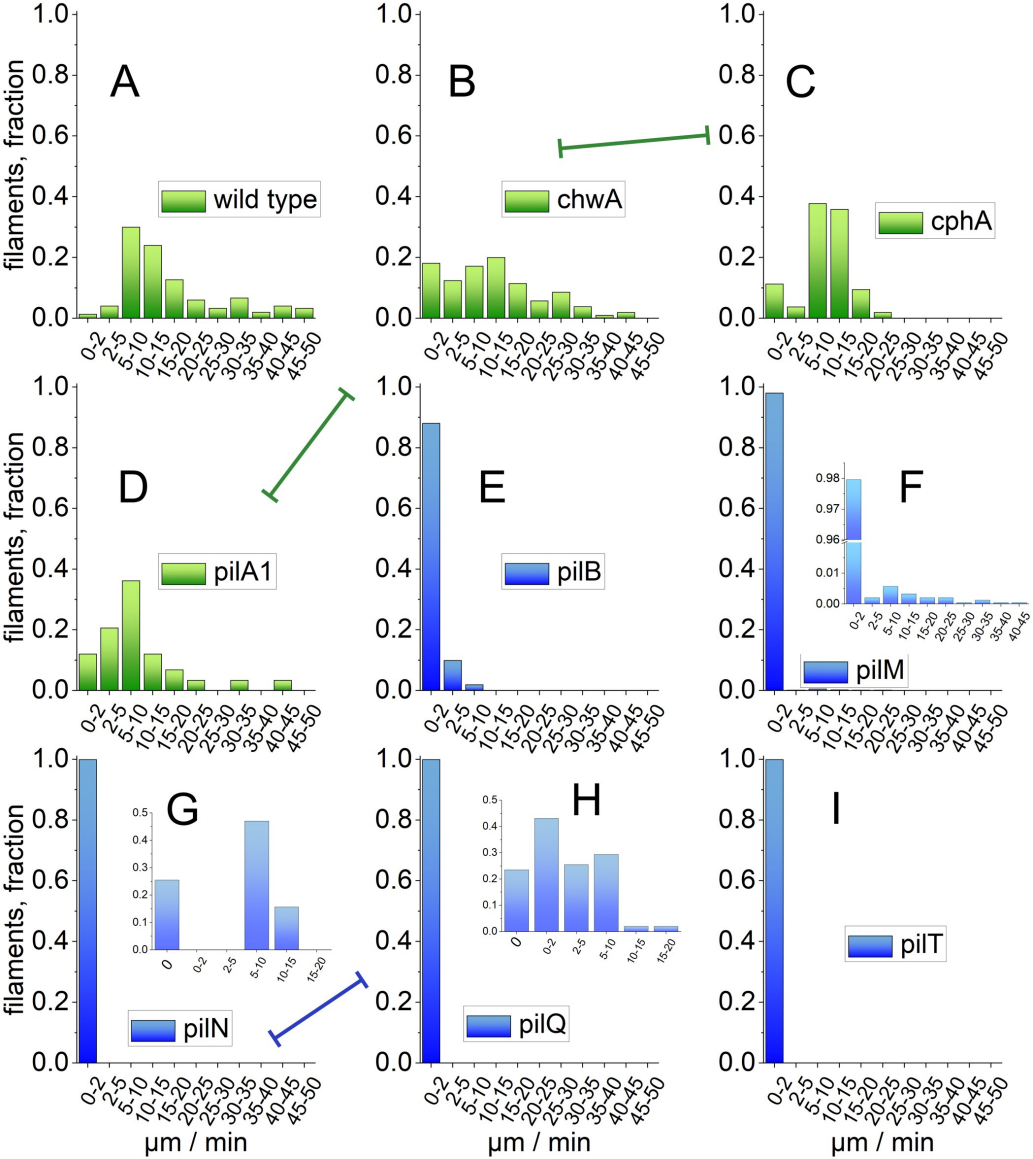
Histograms of motilities of wild type and mutant filaments after Utraturrax treatment on agar surface. Results of wild type (A), *chwA* (B), *cphA* (C) and *pilA1’* are represented by green bars. These strains showed a clear motility on agar. Results of *pilB* (E), *pilM* (F), *pilN* (G), *pilQ* (H) and *pilT* (I) are represented by blue bars. In these strains motility is drastically restricted or zero. The inset in F shows the results of *pilM* with expanded Y-axis. The insets in G and H for *pilN* and *pilQ* are based on 1 h measurements that were calculated back to 1 min for comparison. The 1 h measurement of *pilT* did not indicate any movement and is not shown. The lines with blocked ends are drawn between pairs of mutants that have no significantly different data (B–D, C–D, G–H), those of all other pairs of mutants or wild type are significantly different (Kruskal Wallis test, p < 0.05).

The speed of movement of wild type and mutant filaments was estimated from data obtained during the early stage after Ultraturrax treatment, during which single filaments or few parallel filaments predominate. The distances covered during 1 min were analyzed for 50 to 150 filaments of each strain. During this short time interval, the switching of direction was rare and the measurements are thus more reliable than for longer time intervals. As can be seen in the histogram in Fig 2A, the speed of wild type filaments was variable between 2 and 60 μm / min. The interval between 0 and 2 μm could not be resolved under these conditions. About 30% of wild type filaments were in the range from 5 to 10 μm, the range that contained most filaments. The mutants could be divided into two groups, one group consisting of *chwA*, *pilA1’* and *cphA* which showed slightly reduced wild type like movements (Fig 2B–2E) and the other group comprising *pilB*, *pilM*, *pilN* and *pilQ* which showed drastically reduced movements or no movements. Since none of the data follow a Gaussian distribution, we estimated the significance of differences by the non-parametric Kruskal Wallis ANOVA test [45]. In this way, results from all mutants were compared with each other. All strains were found to be significantly different from each other (p < 0.05) and from the wild type with the exceptions of the three pairs *chwA-cphA*, *chwA-pilA1’* and *cphA-pilA1’*, for which the differences were not significant. This is indicated by the lines between panels in Fig 2B–2D, 2G and 2H. As noted above, the speeds of *chwA*, *cphA* and *pilA1’* were reduced as compared to the wild type. This effect could have been caused by the insertion of the KanR cassette in all three cases, and the possibility that KanR has a similar impact on other mutants must be considered. It is also possible that the motilities of these three mutants are affected by the knockout or by the partial knockout. Note that during these tests all filaments were in Kn free medium.

The mutants *pilB*, *pilM*, *pilN*, *pilQ* and *pilT* exhibited significantly reduced motility. This shows clearly that gliding motility is based on the action of type IV pili as shown for single celled cyanobacteria [10], for Nostocales [8], and for many bacteria [14]. The pilus motor proteins PilB and PilT, the inner membrane pore forming proteins PilM and PilN and the outer membrane core protein PilQ are all required for proper functioning of type IV pili and for gliding motility of *Phormidium lacuna*. However, only *pilT* mutants have completely lost their motility, whereas in all other mutants there was a small fraction of filaments that remained motile or at least slightly motile. In case of *pilB* (Fig 2E), the fraction of motile filaments was ca. 10%. For *pilM*, a fraction of 4% remained motile with a distribution like the wild type. This is shown in the inset of Fig 2F. For *pilN* and *pilQ*, the movement was also measured during 1 h time intervals. In this way, also slow movements were detected (inset of Fig 2G and 2H). Such 1 h measurements were performed with *pilT* as well, but no movement was detected at all.

Fig 1 shows Ultraturrax treated filaments that were kept for 4 h on agar. Wild type filaments aligned parallel to each other as described above, forming bundles of up to 20. Bundles were also formed by *chwA*, *pilA1’* and *cphA*, and no difference to the wild type was observed. With *pilB*, also bundles were formed that could not be distinguished from the wild type. As noted above, only a fraction of 10% of *pilB* filaments seems to move (Fig 2), but the long-term observations in Fig 1 shows that all filaments of *pilB* can move. We confirmed a “wake up” of previously immobile *pilB* filaments when time lapse recordings were re-investigated. *pilM*, *pilN*, *pilQ* and *pilT* did not form bundles, but filaments remained single or crossed over. Since in the pilin mutants there is a clear correlation between lost bundle formation and reduced motility, the type IV pili are required for bundle formation. Bundle formation requires also that filaments recognize each other and that contacts are formed. It is possible that type IV pili play important roles in these processes.

### Motility in liquid medium

A largely unnoticed kind of movement that is different from the gliding movement has been described for Oscillatoriales members [46]. This movement occurs in liquid culture in lateral direction, *i*.*e*. perpendicular to the filament axis. We found such movements when *Phormidium lacuna* filaments were observed with a microscope directly in liquid cultures. The lateral movement was more rapid than the longitudinal gliding movement described above. It could be observed directly without time lapse recordings. After Ultraturrax treatment, some filaments started immediately with lateral movements (S2 Movie). Within few minutes, hedgehog-like bushes were formed, with ends of the filaments pointing away from the center towards the medium. Filaments are apparently connected by an extracellular matrix based on a network of polysaccharide and proteins [47]. In our samples, a matrix could be observed by placing a needle tip into the liquid medium next to filaments. A move of the needle caused a move of the nearby filaments in the same direction, although there was no contact visible between filament and needle. Through the network comprising adjacent filaments and the intermediate extracellular matrix, force could be generated between filaments for lateral movement, without the need for surface contact as in gliding motility.

**Fig 3:**
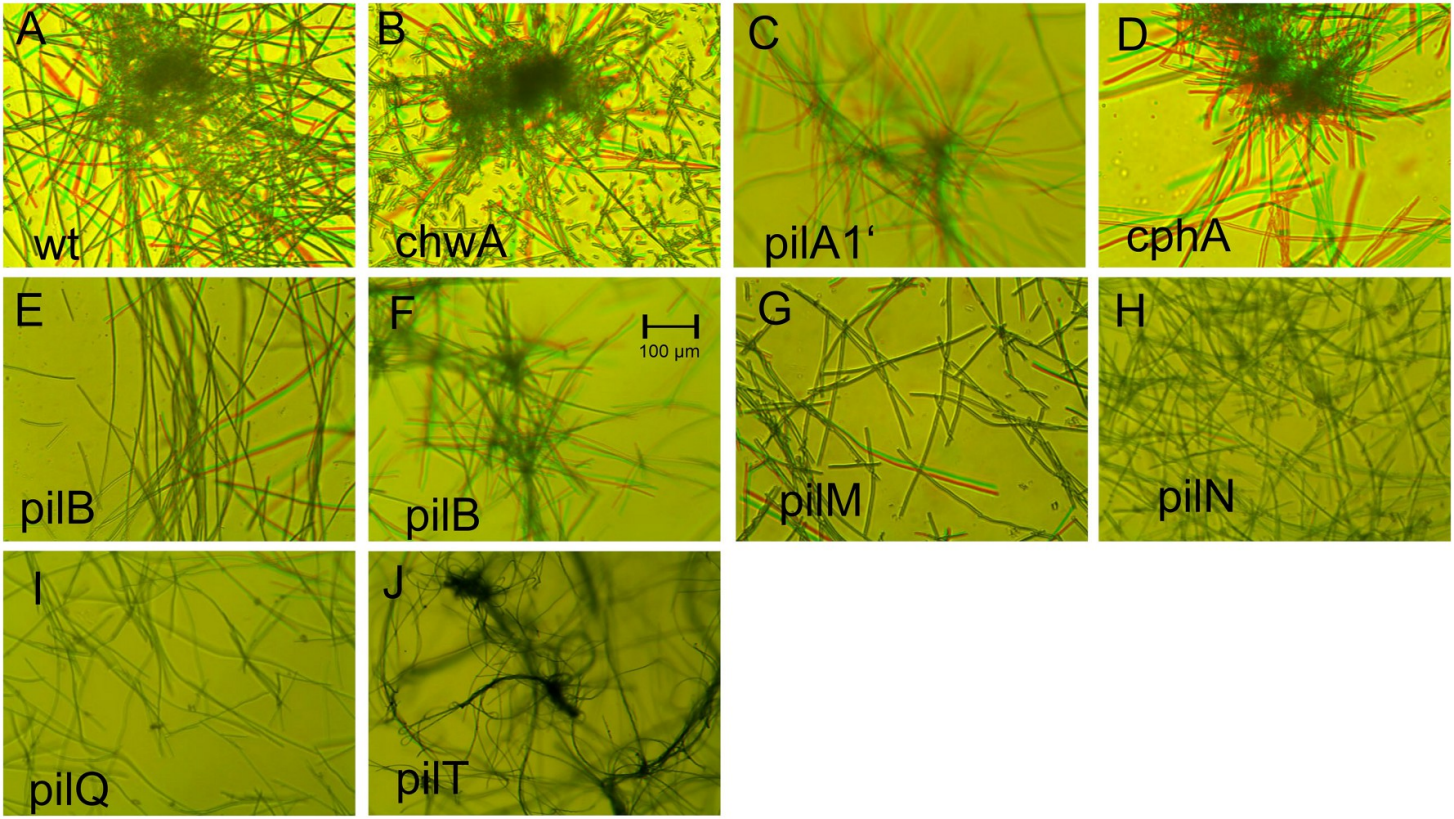
Motility in liquid culture. In each panel, two pictures with red and green false colors are superimposed. The time difference between both was 10 s. Moving filaments are highlighted by red or green color. The name of each mutant is given in the panels. For *pilB* mutants there are two pictures, E and F, with weak and stronger movement, respectively. See also S2–S10 Movies.

For wild type and each mutant, we made recordings of five or more different views and for each view about 5 recordings in series. Each recording was 30 s long. We tried to quantify movement speed or other parameters by different analysis software, but could not obtain reliable results. However, clear differences between strains became evident through qualitative comparisons. Here, we provide selected videos (S2–S10 Movies) and merged images for each strain (Fig 3). The merged images are composed of one picture that is displayed in red and a second one taken after 10 s that is displayed in green. Moving filaments are thus displayed in red or green. Lateral movements of wild type filaments in liquid culture can be seen in S2 Movie and Fig 3A. All filaments made lateral movements, although at a certain time point only a subfraction moved, as seen also in Fig 3A. The *chwA* strain was indistinguishable from the wild type (Fig 3B and S3 Movie). Also, *phrA1’* and *cphA* displayed movements similar to the wild type (Fig 3C and 3D, S4 and S5 Movies). The results of the *pilB* mutant differed qualitatively from one sample to another. In 3 of the 6 sample series, only few filaments moved with slow speed (as in Fig 3E). In the other 3 sample series, almost all filaments moved (as in Fig 3F and S6 Movie), although still less as compared to wild type. The reason for this difference among the *pilB* mutant samples is not known. As in the gliding motility experiments, PilB is required for efficient movement, but dispensable for movement in general. With *pilM* mutants, we also observed that few filaments moved (Fig 3G), but most filaments remained silent (S7 Movie). This result is again equivalent to the gliding motility study where very few filaments showed normal movement. For *pilN*, *pilQ* and *pilT* mutants, no lateral movements were observed (Fig 3H–3J, S8–S10 Movies). The *pil* mutants show clearly that type IV pili are required for lateral movement in liquid. The mechanism for lateral motility must be based on connections between filaments *via* an extracellular network.

### Photophobotaxis

**Fig 4:**
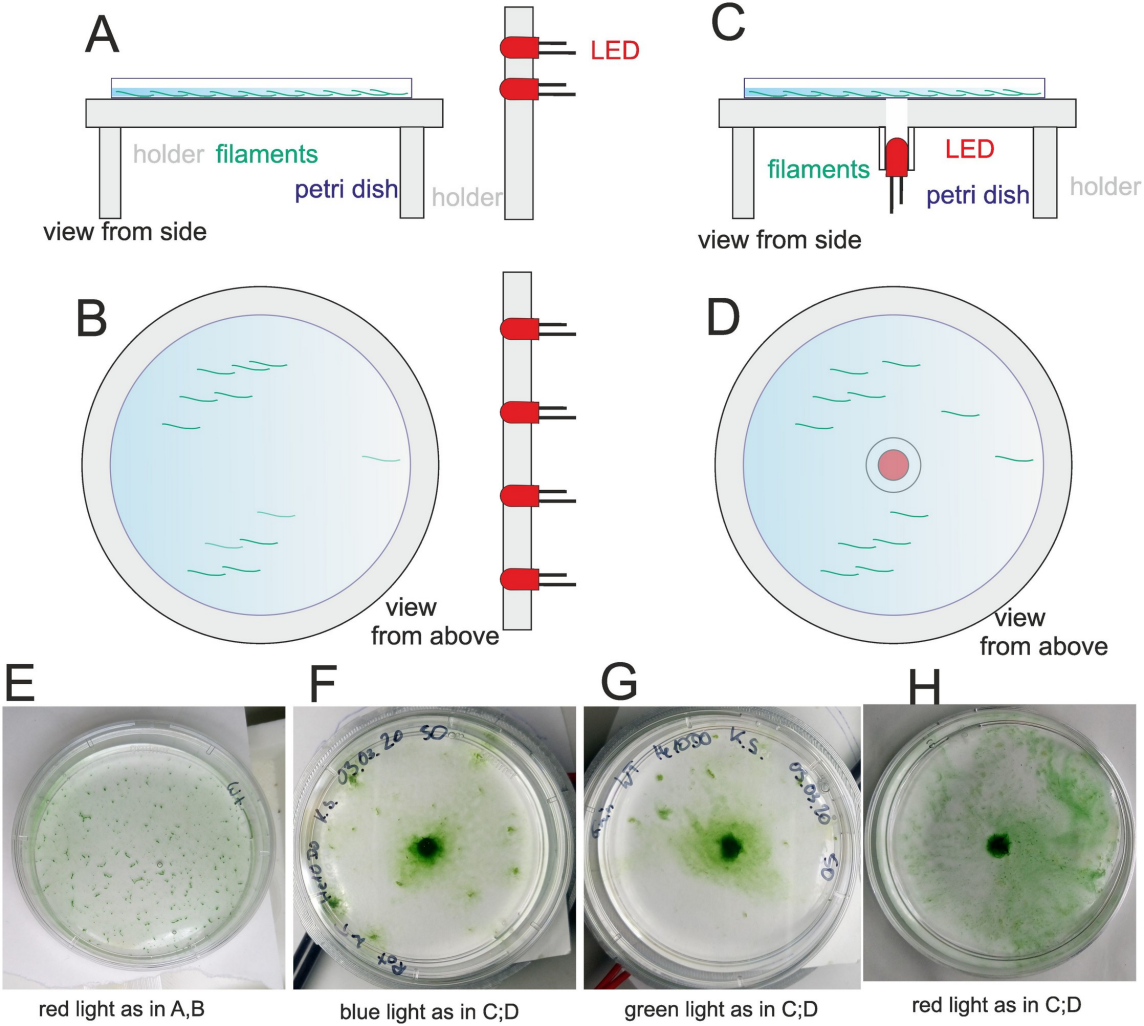
Setup of phototaxis experiments with unilateral irradiation (from the side, A, B) or vertical irradiation (C, D) and examples for irradiations with red light from the side (E) or vertical blue (F), green (G) or red light (H) from below. Light intensities were 25 μmol m^-2^ s^-1^ for blue (450 nm) and green (550 nm) and 15 μmol m^-2^ s^-1^ for red (655 nm) light. The duration of irradiation was 2 d.

In the phototactic response, motile organisms move either towards the light or away from it. This response can most often be induced by unilateral light, *i*.*e*. by light that hits the organism within the area of possible moving directions. We performed many trials with *Phormidium lacuna* to induce phototactic movement by unilateral light (as outlined in Fig 4A and 4B) but the filaments remained equally distributed in the Petri dish (as in Fig 4E). Neither the use of agar plates nor the use of different irradiation wavelengths nor the variation of intensities resulted in a directional response. However, in time lapse studies with *Phormidium filaments* we observed that filaments sometimes gather in the white microscope light that hit the sample from below. An example for this effect is shown in S1 Movie. We therefore suggested that light projected vertically to the movement area can induce light-directed movement of *Phormidium lacuna*. In our subsequent studies, *Phormidium lacuna* filaments were irradiated with light emitting diodes (LEDs) from below. In standard assays, 5 cm Petri dishes (without agar) that were filled with 8 ml Ultraturrax-treated filaments in f/2 medium were placed on a holder in which a light emitting diode shines light through a short tunnel on the specimen from below, at the position of the center of the Petri dish (Fig 4C and 4D). We observed that red light, blue light and green light of around 5–50 μmol m^-2^ s^-1^ induce a clear gathering of wild type filaments towards the light in the center (Fig 4F–4H). Gathering could be observed after several hours. During 2 d the effect became stronger. Thus, the movement of *Phormidium lacuna* in light follows rather a light-intensity gradient than the light direction and behaves therefore different from single celled cyanobacteria. Directional effects by vertical light have been described for other organisms such as the alga *Euglena gracilis* [48] or *Phormidium uncinatum* [27, 49]. Responses towards unilateral light and responses towards light gradients induced by vertical light have been termed phototaxis [48]. Responses towards vertical light have also been termed photophobotaxis [27], because the organisms avoid darkness. In order to emphasize the difference between the responses of *Phormidium lacuna* and of single-celled cyanobacteria, we use the term photophobotaxis here. It should be stressed that this movement takes place in Petri dishes with or without agar and that the majority of photophobotaxis experiments were performed without agar.

**Fig 5:**
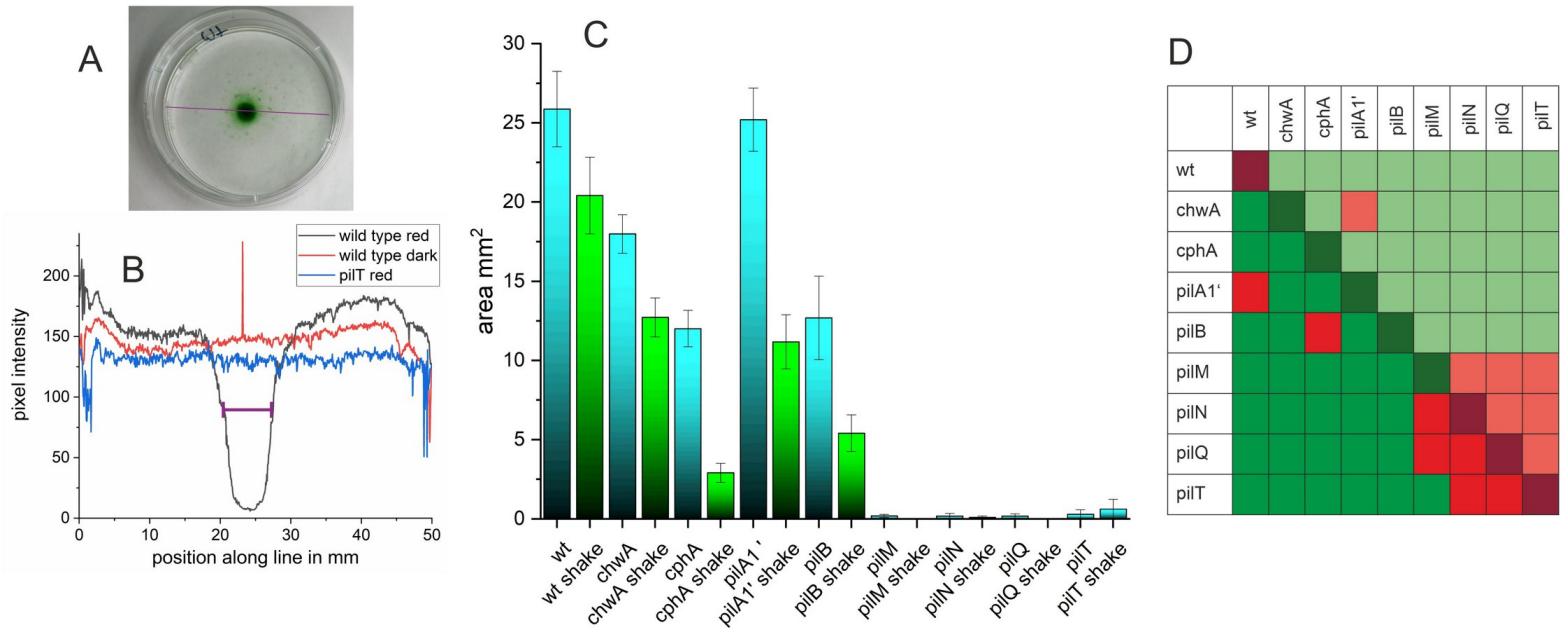
Phototaxis experiments with *Phormidium lacuna* wild type and various mutants. (A) Petri dish with wild type filaments after 2 d irradiation with central LED from below. Pixel intensities along the violet line are taken for calculation of the diameter. (B) Typical profiles of pixel intensities as outlined in A for wild type after red-light illumination, wild type without illumination and *pilT* after red-light illumination. The estimated diameter for wild type red is given by violet distance (C) Mean area after 2 d in red light from 5 mm wide LED, before (blue bars) and after (green bars) shaking. (D) Kruskal Wallis significance test, comparison of all samples with each other. The off-diagonal matrix elements in the lower left show the comparisons between samples before shaking, the corresponding elements in the upper right show the comparisons between samples after shaking, and the diagonal elements show the comparisons between each sample before and after shaking. Green squares indicate significant differences with p < 0.05, and red squares indicate non-significant differences.

Comparative analyses of wild type and mutants were performed with red light at a light intensity of 15 μmol m^-2^ s^-1^ for 2 d. Thereafter, a photograph was recorded from the Petri dish. We routinely made a second photograph after a gentle shaking of the Petri dish. The diameter of the covered circle was measured between the two positions of half maximal pixel intensity along a line through the center of the circle (Fig 5A and 5B), this value was used to calculate the circle area. The area value stands as a measure for the magnitude of the photophobotactic response. Shaking reduced the size of the densely covered area. In this way, the data distinguish between all filaments that have moved to the center and those filaments that were more tightly bound to the surface. Shaking did not increase the error values to a relevant extent (Fig 5C), which supports the reliability of the approach.

The circle area of the light beam is 20 mm^2^ and in a clear response the entire area is completely covered with filaments. We experienced that a response could result in a covered area even larger than 20 mm^2^, and any smaller covered areas were also often observed. In samples where no accumulation in the center was obvious, as in the dark control or *pilT*^*-*^ examples shown in Fig 5B, we set the area value to 0. The data processing showed a broad distribution of covered areas (S1 Fig) for wild type, *cphA*, *pilA1’*, *chwA*, *pilB*. The ranges were from no response to intermediate and strong responses with covered areas above 25 mm^2^. The mean values are presented in Fig 5C. We estimated the significances of differences between all possible pairs of samples according to Kruskal and Wallis [45]. Significance of sample pairs before shaking is presented by colored squares in the lower left triangle of Fig 5D, the pairs of samples after shaking are presented by the colored squares in the upper right triangle of Fig 5D and the significance of each sample before and after shaking are given by the colored squares in the diagonal in of Fig 5D.

The wild type covered an average area of 25 mm^2^. Shaking reduced the wild type area to a mean value of 20 mm^2^, i.e. the irradiated area. With ca. 18 mm^2^, the mean covered area of *chwA* was reduced as compared to the wild type; shaking reduced the area to 12 mm^2^. We assume again that the reduced response of *chwA* vs. wild type results from the KanR cassette. In case of *pilA1‘*, the covered area was 25 mm^2^, the same as in the wild type. The mutation does not affect the magnitude of photophobotaxis. If the KanR effect would be the same as in the *chwA* mutant, the partial loss of *pilA1’* would compensate for this effect.

The response of *cphA* was further reduced compared to wild type, *chwA* and *pilA1’*. Before and after shaking the covered areas were 12 mm^2^ and 2 mm^2^, respectively. These low values of *cphA* imply that in the wild type, CphA modulates photophobotaxis or acts as a photoreceptor for this reponse.

The *pilB* mutant had a covered area of 12 mm^2^ and an area of 5 mm^2^ after shaking. The pattern is comparable with *cphA*. The cause for reduction could be the reduced motility of *pilB*, but also a reduced adhesion, which could result from partially defective type IV pili.

The other pilin mutants *pilN*, *pilM*, *pilQ and pilT* had no detectable phototactic response. Only in exceptional cases, a response was obtained. That for a photophobotactic response the integrity of type IV pili is required is no surprise, as photophobotaxis without motion is not possible.

## Discussion

We describe here for the first time mutant effects on the motility of a member of the Oscillatoriales. *Phormidium lacuna* is an ideal model cyanobacterium for such studies, because natural transformation is established and because the filaments are continuously in motion. The motility of the genera *Oscillatoria* or *Phormidium* have been scientifically investigated since the 19^th^ century [6, 49–52]. Cellular and molecular studies on motility of cyanobacteria have, however, concentrated on the model organism *Synechocystis* sp. PCC 6803 [10, 53], a unicellular species. Few experiments followed with *Nostoc punctiforme* (Nostocales) and *Synechococcus elongatus* (single celled) [12]. There are three major differences between motilities of *Phormidium lacuna* and single celled cyanobacteria. (i) The gliding motility of *Phormidium lacuna* is characterized by continued change of direction, combined with bending movements, and thus essentially different from the straight movement of single celled cyanobacteria. (ii) *Phormidium lacuna* makes lateral motions in liquid medium (S2–S10 Movies), such motions are not known for single celled species. (iii) Finally, the photophobotactic movement of *Phormidium lacuna* is induced by vertical light, in contrast to the movement towards unilateral light in phototaxis of single celled species. The mechanisms of light perception and detection of light direction must be completely different between these species. Although type IV pili are widely distributed in cyanobacteria and present in *Phormidium lacuna*, it was initially not clear whether these pili are involved in motions of *Phormidium lacuna*. Lateral motion, independent of surface contacts, is rarely described in cyanobacteria. The direction of lateral movement is opposed to the longitudinal direction in gliding motility. Because *pilM*, *pilN*, *pilQ* and *pilT* were neither motile in gliding motility nor in lateral movements, both kinds of movement must be driven by type IV pili. For lateral motility, this implies that the outer ends of the pili are connected with the extracellular matrix or with pili of other cells, in order to generate force for the motions. Alternatively, another motile structure which is dependent on intact type IV pili, could perform lateral movement. What evolutionary advantage can be found behind lateral motions in liquid? The back and forth movements do not allow the filaments to reach another place. However, filaments join neighboring filaments and form aggregates, and this kind of biofilm formation could protect against predators. The counterpart of these aggregates are the aligned filaments on agar that form leaflike structures (Fig 1). Also in this case, the getting together could result in protection against predators.

The results from all three assays, gliding motility, lateral motions in liquid and photophobotaxis indicate that PilM, PilN, PilQ and PilT are essential for the function of the type IV pili—without these any kind of motion is inhibited. PilN and PilM are pore forming components in the inner membrane and PilQ is involved in pore forming at the outer membrane. PilT is an ATPase for retraction of the pilus. Clearly, each of the tested components of the Type IV pilus apparatus and its assembly components are required for motility. We would like to stress here that it is very unlikely that the immobile phenotypes result from second site mutations. We have performed many *Phormidium* transformations in which other sites were targeted. The immobile phenotype was never observed.

In *pilB* mutants motility is only partially blocked in the three motion assays. PilB is the ATPase for pilus expelling. The PCR results indicate complete segregation of the interrupted gene. As the other mutants, this strain was kept continuously on Kn medium until it was used for experiments. There is thus no reason to assume that wild type *pilB* copies are still abundant in the cells. In recent RAST [54] annotations we found four proteins with weak homology to the PilB ATPase domain in the genome of *Phormidium lacuna*. Within ca. 300 amino acids of the ATPase region, PilB and the homologous proteins revealed ca. 20% identity. These proteins could replace PilB in the *pilB* mutant, although the N-terminal T2S domain of PilB is lacking in these homologous proteins. Alternatively, another yet unsequenced *pilB* could replace the regular one, but this option is unlikely. We have sequenced draft genomes of altogether 5 strains, and more than 90% of each genome are completely sequenced. A second *pilB* was found in none of the strains.

For *pilA1’* we obtained only partially segregated disruption strains, which were almost as motile as the wild type. In case of *pilD* it was even not possible to select strains in which the disrupted gene is partially segregated. The loss of PilD in *Synechocystis* sp. PCC 6803 is lethal because PilA1 prepilin proteins accumulate in the membranes thereby inhibiting synthesis of photosystem II proteins [44]. The same effect could explain why *pilD* of *Phormidium lacuna* is lethal. A *pilA1* mutant would, however, not cause such effects. It is difficult to understand why *Phormidium lacuna* cells cannot survive without PilA1. *Phormidium lacuna* has 2 homologs of PilA2 (WP_140409148, 30% identities of overlapping region, WP_087711780.1, 31%), but no other PilA1 homolog. For *Phormidium lacuna* we could imagine that misassembly of the pilus due to lack of PilA1 results in accumulation of PilA2 proteins in the plasma membrane and in cell death of completely segregated *pilA1* mutants.

The *cphA* mutant shows motility on agar comparable with the wild type, *chwA* and *pila1*. The photophobotaxis response of *cphA* was, however, clearly diminished as compared to the other three strains. CphA operates either as a photophobotaxis photoreceptor in *Phormidium lacuna* or as a modulating photoreceptor that acts on the photophobotaxis signal transduction pathway. Typical phytochromes are red light sensors and our relevant phototaxis experiments have been performed in red light. The weak phototaxis response in the *cphA* mutant must be regulated by a different photoreceptor. Phototaxis of *Phormidium lacuna* is observed in blue, green and red light. Therefore, it is very likely that more than one single photoreceptor modulates phototaxis. The role of CphA in photophobotaxis is another major difference between *Synechocystis* sp. PCC 6803 and *Phormidium lacuna*, since knockout mutants of the homologous Cph1 in *Synechocystis* were not affected in phototaxis. CphA could also act indirectly on photophobotaxis, for example if photophobotaxis is mediated through photosynthesis, as it has been hypothesized for filamentous cyanobacteria [13, 27]. Further mutant experiments are required to clarify the photophobotaxis photoreceptor situation in *Phormidium lacuna*.

What is the mechanism behind *Phormidium lacuna* photophobotaxis? Random movement in the dark and stop of movement by surface attachment (Fig 5) in the light could explain a gathering in the light zone. Surface attachment would be part of this mechanism. However, the time lapse studies (as in S1 Movie) do not support such “move in the dark or attach in the light” response, because movement of wild type filaments and filaments of motile mutants is observed in the microscope light. We therefore also believe that the sticking to the surface is not part of the phototactic response. A simple model for photophobotaxis of *Phormidium lacuna* could be as follows: Filaments move in darkness and in light in random directions, but the moving direction switches more often when they leave the light. In this way, filaments would finally accumulate in the light. For a detailed investigation of the mechanism of light sensing, experiments on an infrared microscope with which the movements along light—dark transition can be observed are desired. We also got the impression that stray light induces a gathering response towards the high light intensities (see e.g. bundle in S1 Movie). The lack of phototaxis in unilateral light is at contrast with such an observation. Although details on the mechanism need to be investigated, it is clear that the photophobotaxis of *Phormidium lacuna* differs in many aspects from the phototaxis of *Synechocystis* sp. PCC6803 or other single-celled cyanobacteria.

## Supporting information

S1 Fig PCR results to characterize insertional mutagenesis, agarose gel electrophoresis. (PDF)

S2 Fig Histogram of photophobotaxis. (PDF)

S1 Raw images (PDF)

S1 Table Primers for cloning integration mutants and for detection. (PDF)

S1 Movie Movement of *Phormidium lacuna* wild type filaments after Ultraturrax treatment on agar, time lapse with 1 image every min; 10 x objective. (MP4)

S2 Movie Movement of *Phormidium lacuna* wild type filaments in liquid medium after Ultraturrax treatment; 10 x objective. (MP4)

S3 Movie Movement of *Phormidium lacuna chwA* filaments in liquid medium after Ultraturrax treatment; 10 x objective. (MP4)

S4 Movie Movement of *Phormidium lacuna cphA* filaments in liquid medium after Ultraturrax treatment; 10 x objective. (MP4)

S5 Movie Movement of *Phormidium lacuna pilA1’* filaments in liquid medium after Ultraturrax treatment; 10 x objective. (MP4)

S6 Movie Movement of *Phormidium lacuna pilB* filaments in liquid medium after Ultraturrax treatment; 10 x objective. (MP4)

S7 Movie Movement of *Phormidium lacuna pilM* filaments in liquid medium after Ultraturrax treatment; 10 x objective. (MP4)

S8 Movie Movement of *Phormidium lacuna pilN* filaments in liquid medium after Ultraturrax treatment; 10 x objective. (MP4)

S9 Movie Movement of *Phormidium lacuna pilQ* filaments in liquid medium after Ultraturrax treatment; 10 x objective. (MP4)

S10 Movie Movement of *Phormidium lacuna pilT* filaments in liquid medium after Ultraturrax treatment; 10 x objective. (MP4)
