# Supplementary material for: The involvement of type IV pili and the phytochrome CphA in gliding motility, lateral motility and photophobotaxis of the cyanobacterium Phormidium lacuna
Source: PLoS One. 2022 Jan 27;17(1):e0249509. doi: 10.1371/journal.pone.0249509 (PMC8794177; doi:10.1371/journal.pone.0249509)
Supplement: S1 Fig — (PDF) [file pone.0249509.s001.pdf]

Supplemental Figure S1, please find common legend after the last panel.

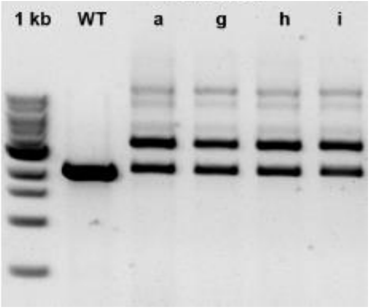

Wild type (WT) and different *pilA* mutants (a,g,h,i), PCR with “inner primers”

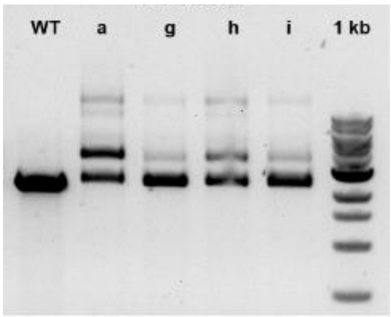

Wild type (WT) and different *pilA* mutants (a,g,h,i), PCR with “outer primers”

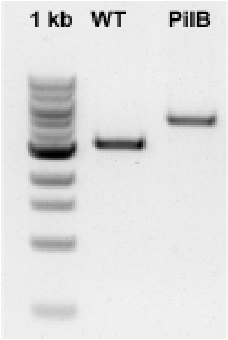

Wild type (WT) and *pilB* mutant (PilB), PCR with “inner primers”

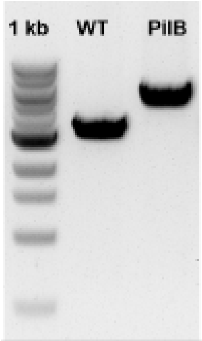

Wild type (WT) and *pilB* mutant (PilB), PCR with “outer primers”

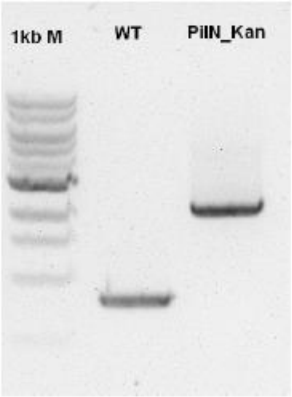

Wild type (WT) and *pilN* mutant (PilN_Kan), PCR with “inner primers”

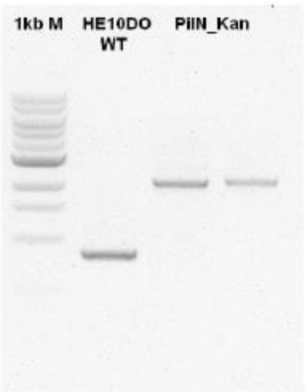

Wild type (HE10DO WT) and *pilN* mutant (PilN_Kan), PCR with “outer primers”

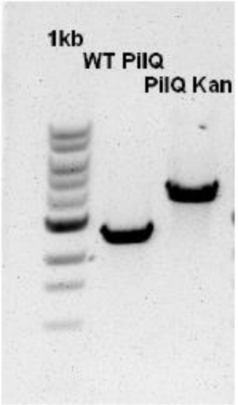

Wild type (WT PilQ)and *pilQ* mutant (PilQ KanR), PCR with “inner primers”

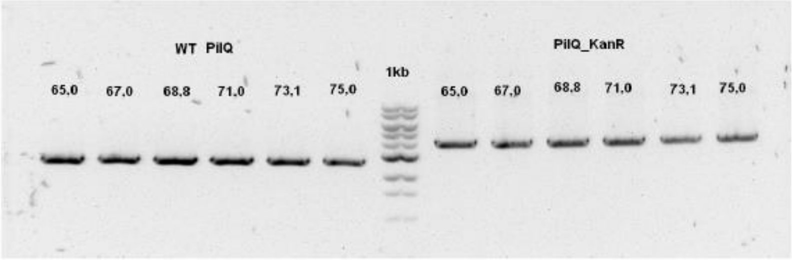

Wild type (WT PilQ) and *pilQ* mutant (PilQ KanR), PCR with “outer primers”. Six PCR products on each side with increasing annealing temperatures as indicated (°C)

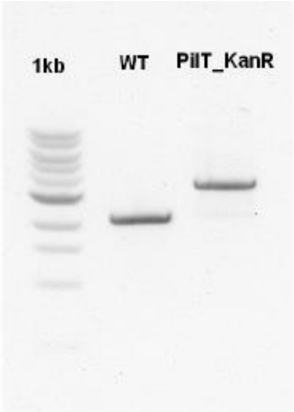

Wild type (WT)and *pilT* mutant (PilT KanR), PCR with “inner primers”

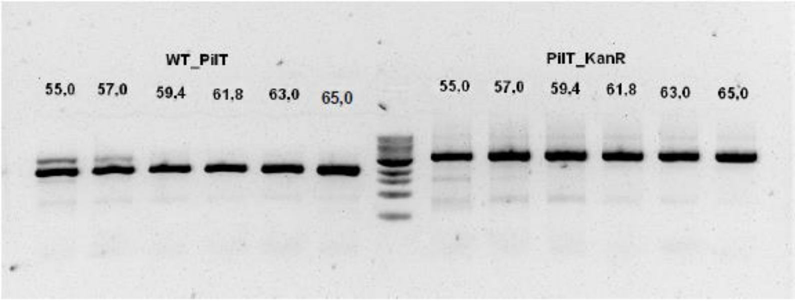

Wild type (WT PilT)and *pilQ* mutant (PilT KanR), PCR with “outer primers”. Six PCR products on each side with increasing annealing temperatures as indicated (°C)

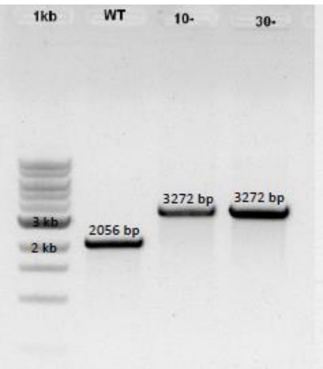

Wild type (WT) and two *cphA* mutants (10- and 30-), PCR with “inner primers”

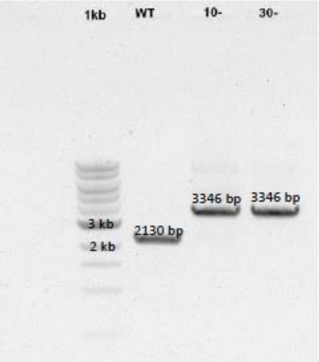

Wild type (WT) and two *cphA* mutants (10- and 30-), PCR with “outer primers”

Supplemental Figure 1. PCR results to characterize insertional mutagenesis, agarose gel electrophoresis

Each panel shows one agarose gel with PCR products from wild type and a mutant. One lane on each gel is the 1 kb DNA ladder. The “inner primers” and “outer primers” are given in Supplemental Table 1.
