# Supplementary material for: The involvement of type IV pili and the phytochrome CphA in gliding motility, lateral motility and photophobotaxis of the cyanobacterium Phormidium lacuna
Source: PLoS One. 2022 Jan 27;17(1):e0249509. doi: 10.1371/journal.pone.0249509 (PMC8794177; doi:10.1371/journal.pone.0249509)
Supplement: S2 Fig — (PDF) [file pone.0249509.s002.pdf]

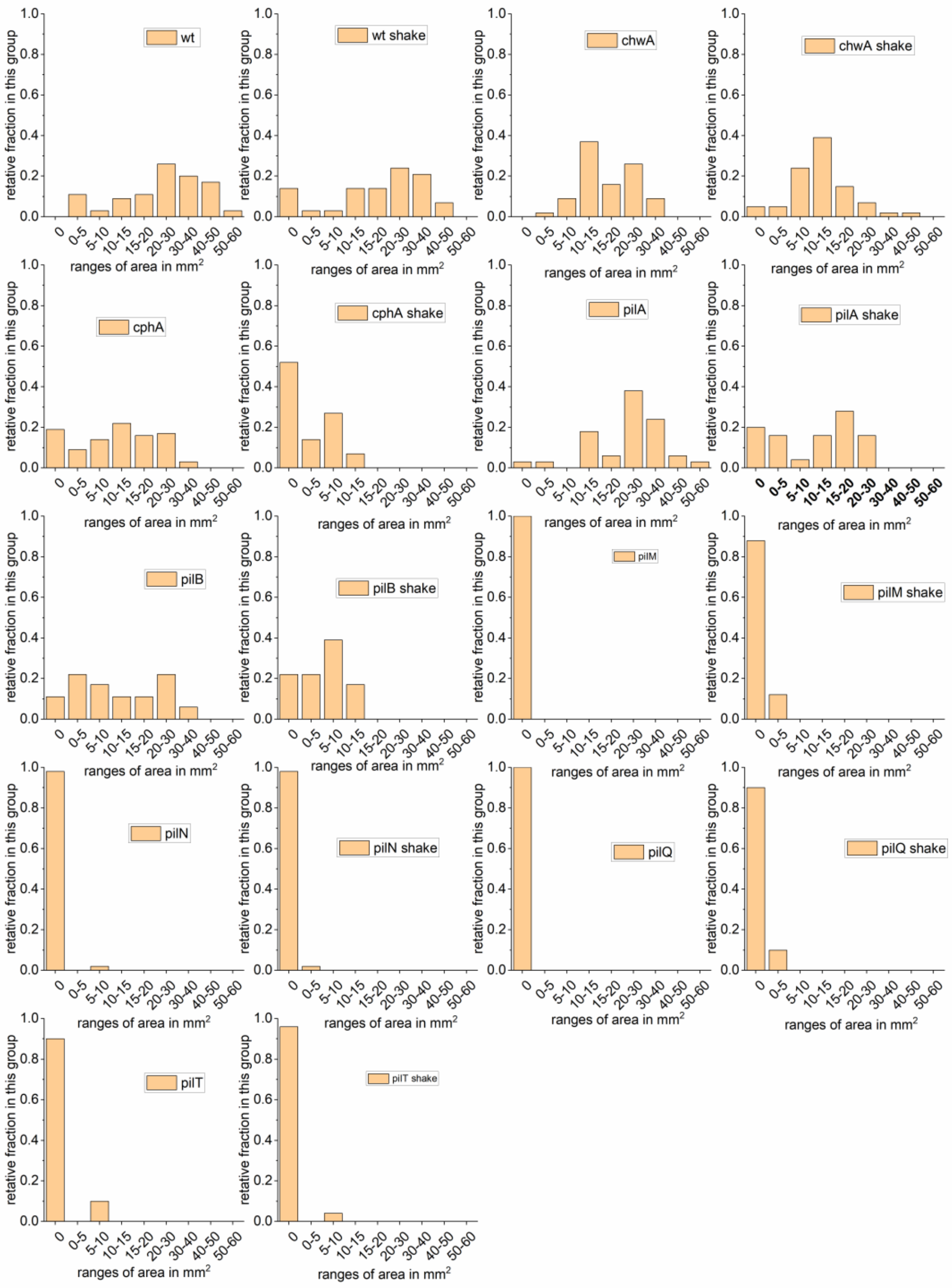

Supplemental Figure 2, photophobotaxis response, histogram presentation. Same data as in Figure 5
