# Supplementary material for: The involvement of type IV pili and the phytochrome CphA in gliding motility, lateral motility and photophobotaxis of the cyanobacterium Phormidium lacuna
Source: PLoS One. 2022 Jan 27;17(1):e0249509. doi: 10.1371/journal.pone.0249509 (PMC8794177; doi:10.1371/journal.pone.0249509)
Supplement: S1 Raw images — (PDF) [file pone.0249509.s003.pdf]

PCR with wild type (WT) sample and different pilA mutants (a, g, h, i) sampel, using "inner primers" (left lanes) and using "outer primers" (right lanes).

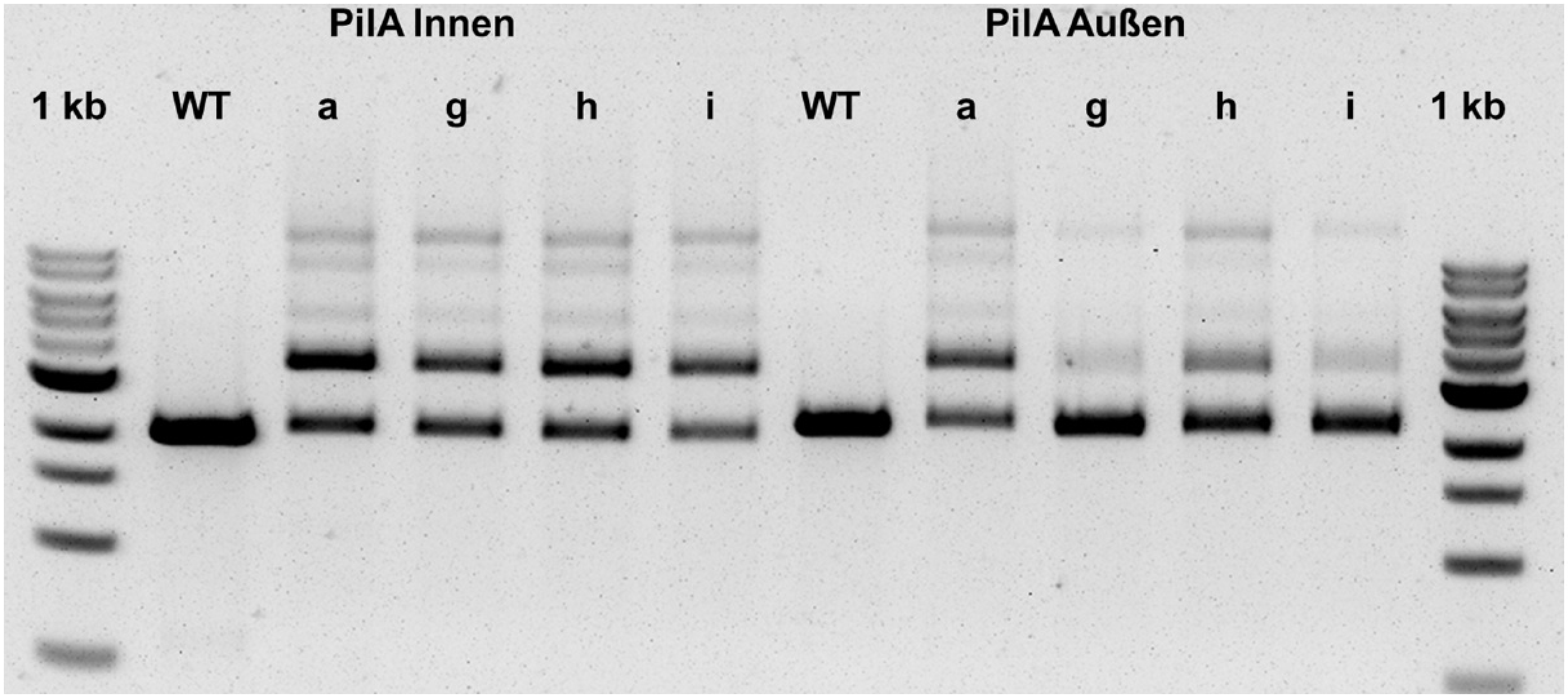

Picture was taken with Intas GDS

PCR with wild type (WT) sample and pilB mutant (pilB) sampel, using "inner primers".

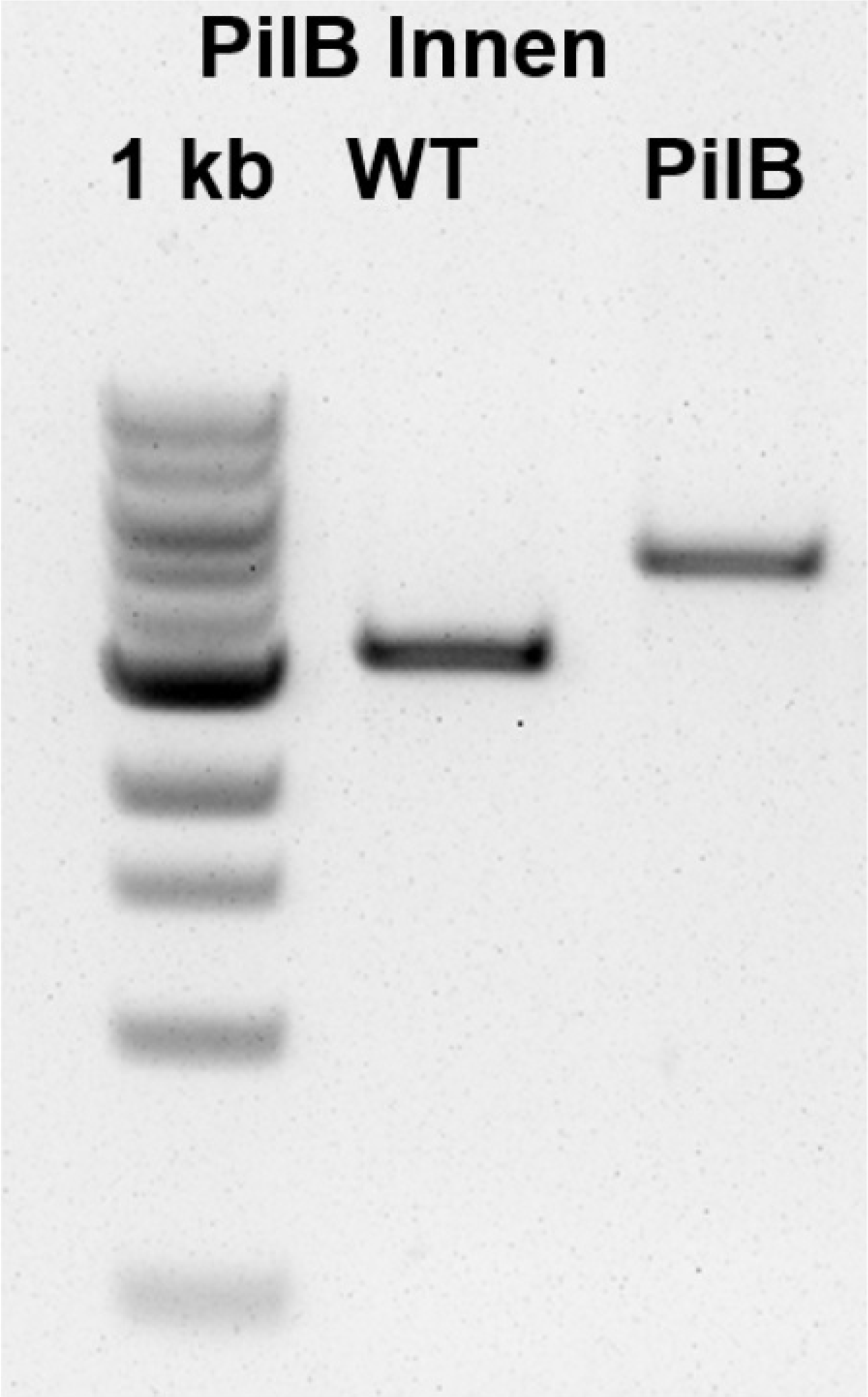

Picture was taken with Intas GDS

PCR with wild type (WT) sample and pilB mutant (pilB) sampel, using "outer primers".

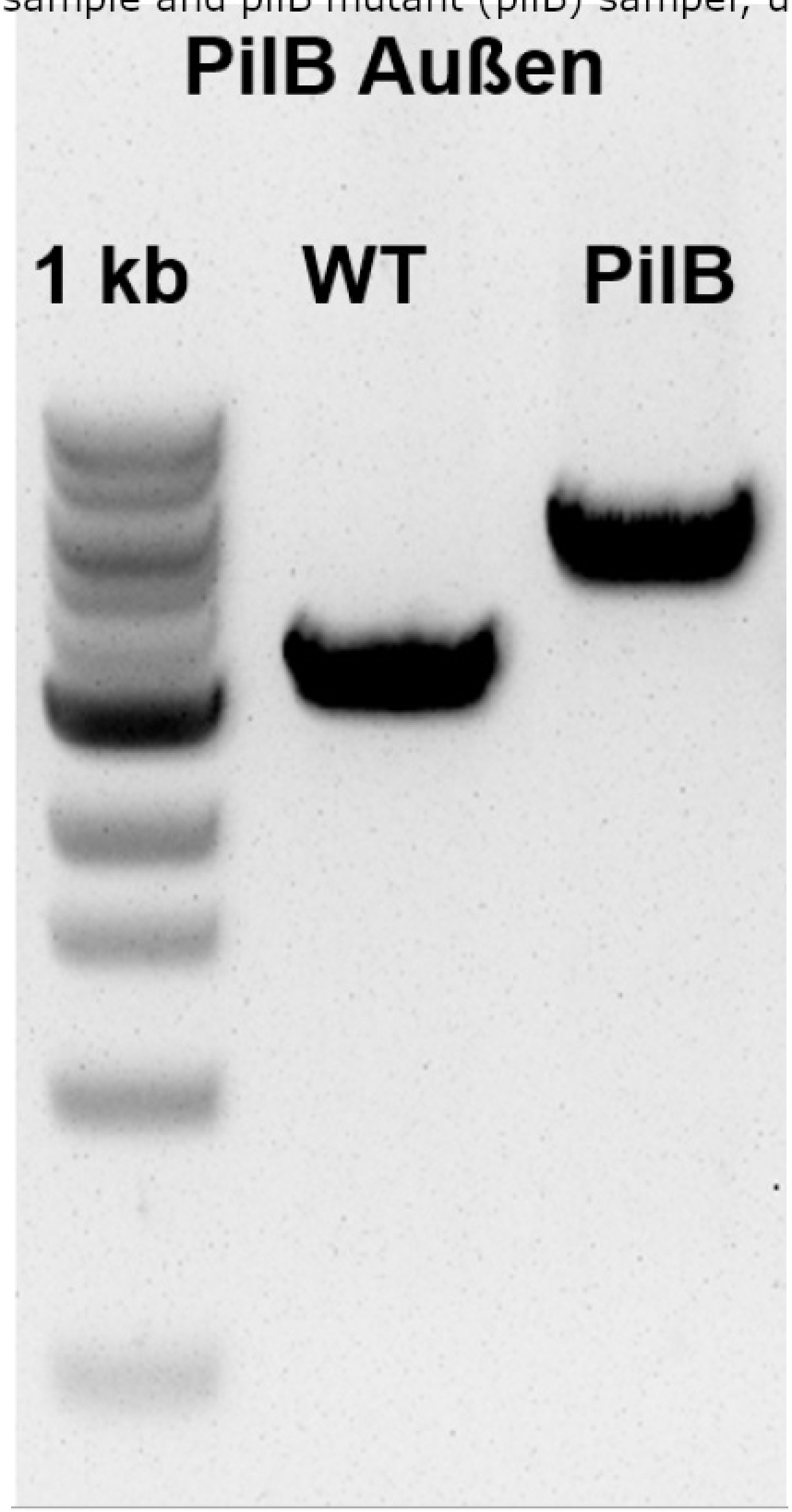

Picture was taken with Intas GDS

PCR with wild type sample (WT) and pilN mutant (pilN_Kan) sample, using "inner primers"

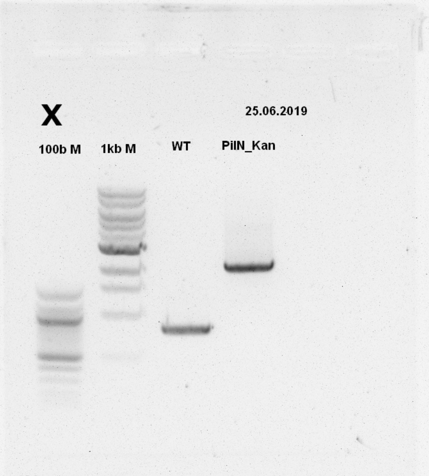

Picture was taken with Intas GDS

PCR with wild type sample (He10DO WT) and pilN mutant (pilN_Kan) sample, using "outer primers"

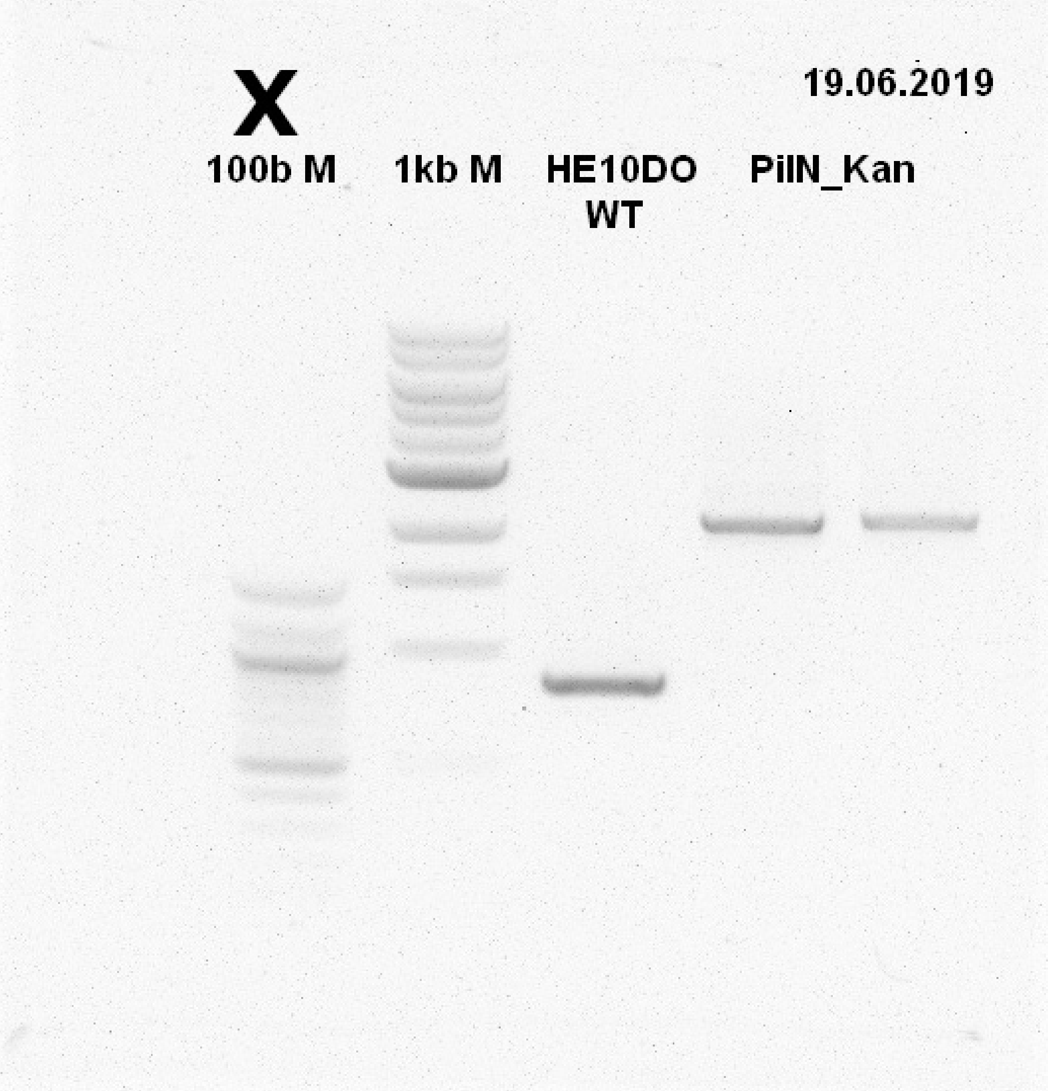

Picture was taken with Intas GDS

PCR with wild type (WT PilQ) sample and pilQ mutant (PilQ KanR) sample, using "inner primers".

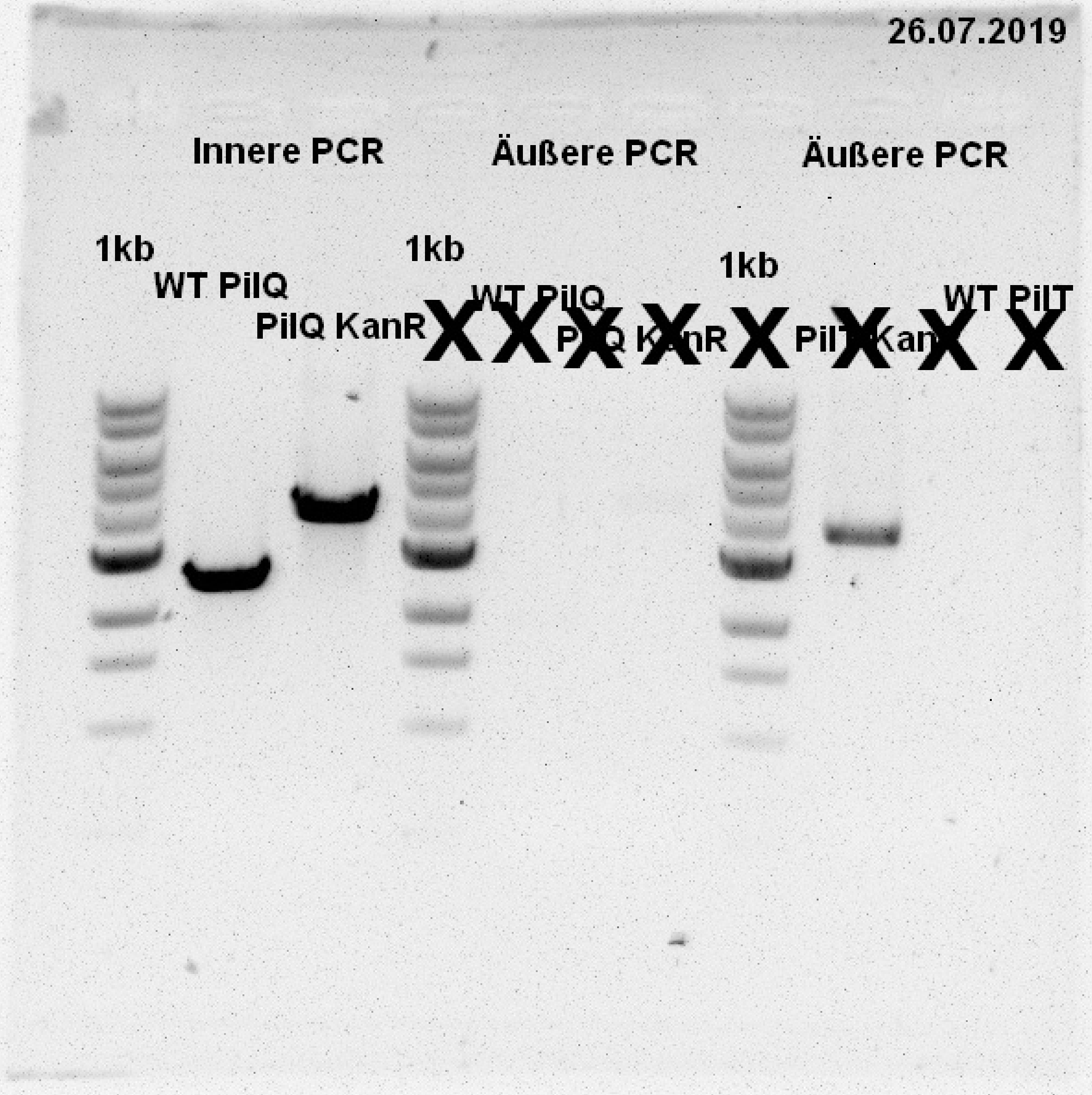

Picture was taken with Intas GDS

PCR with wild type (WT PilQ) sampels and PilQ mutant (PilQ KanR) sampels, using "outer primers". Six PCR products on each side with increasing annealing temperatures as indicates (°C)

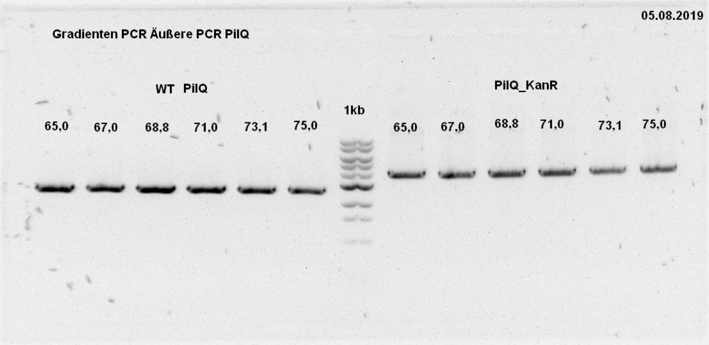

Picture was taken with Intas GDS

PCR with Wild type (WT) sample and PilT mutant (PilT_KanR,) sample, using "inner primers"

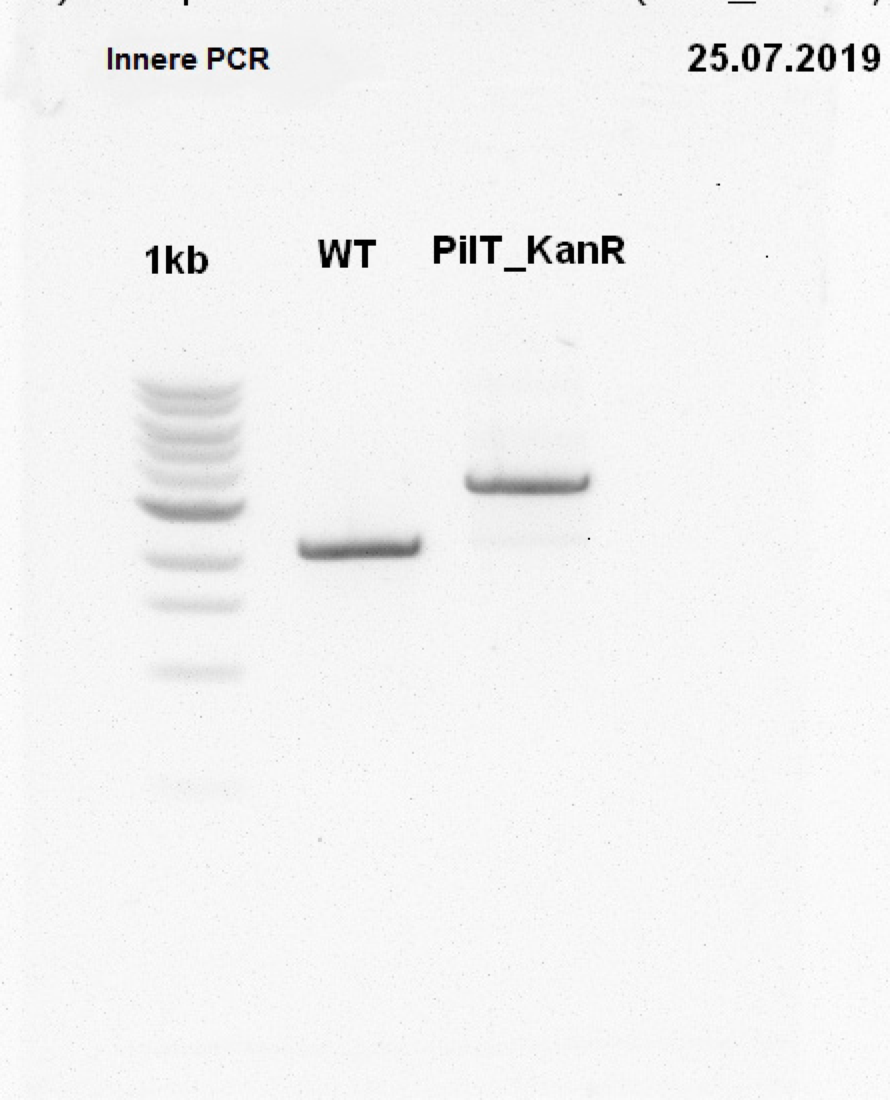

Picture was taken with Intas GDS

PCR with wild type (WT-PilT) sample and pilT mutant sample (pilT_KanR), using "outer primers".
Six PCR products on each side with increasing annealing tempratures as indicated(°C)

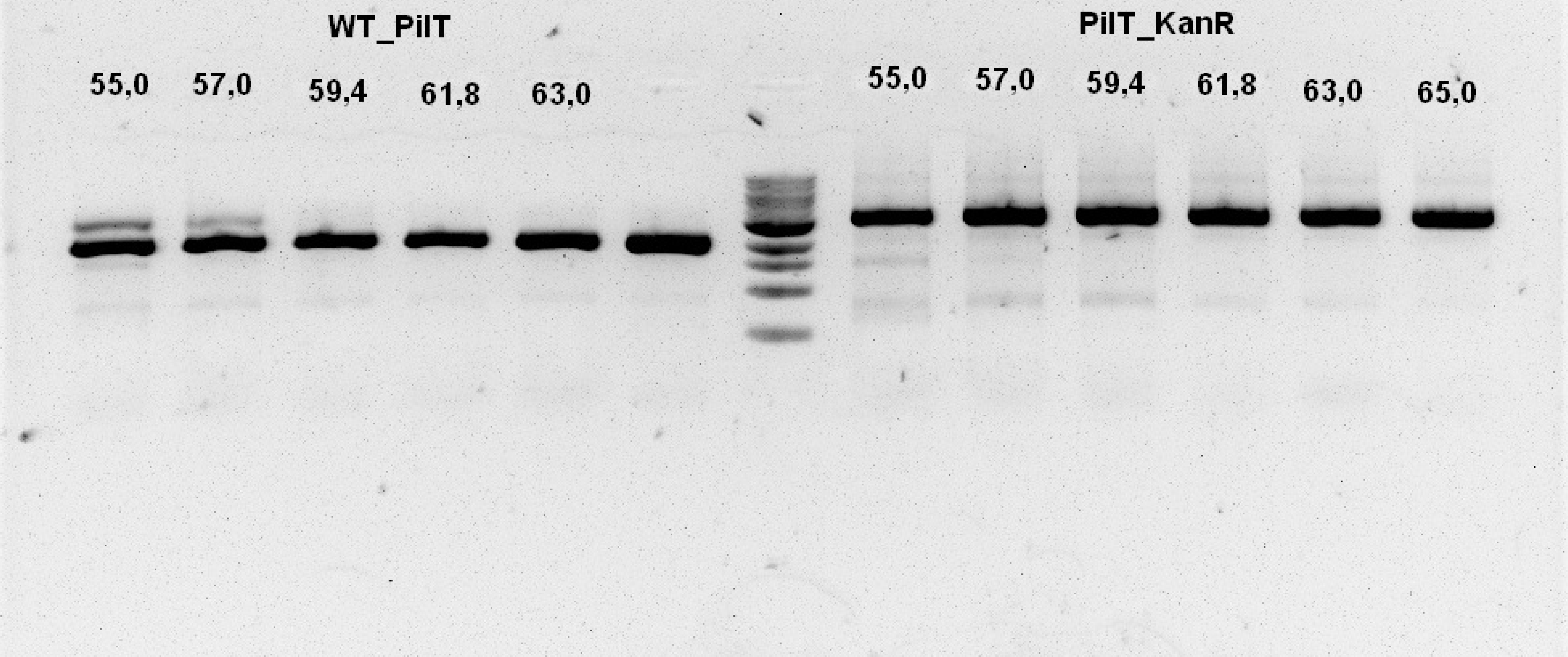

Picture was taken with Intas GDS

PCR with wild type (WT) sampel and two cphA mutant sampels (10- and 30-), using "inner primers".

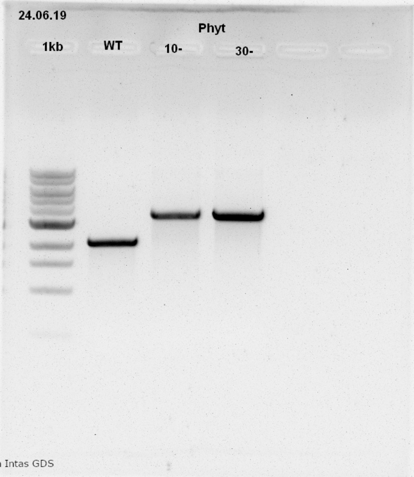

Picture was taken with Intas GDS

PCR with wild type (WT) sampel and two cphA mutant sampels (10- and 30-), using "outer primers".

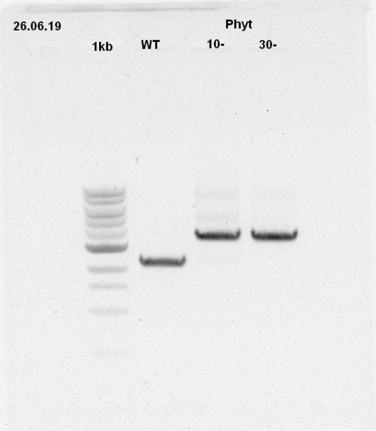

Picture was taken with Intas GDS
