# Supplementary material for: The involvement of type IV pili and the phytochrome CphA in gliding motility, lateral motility and photophobotaxis of the cyanobacterium Phormidium lacuna
Source: PLoS One. 2022 Jan 27;17(1):e0249509. doi: 10.1371/journal.pone.0249509 (PMC8794177; doi:10.1371/journal.pone.0249509)
Supplement: S1 Table — (PDF) [file pone.0249509.s004.pdf]

Lamparter et al.

Supplemental material

Supplemental Table 1. Primers for cloning integration mutants and for detection. Each pair of primers is given in subsequent lines. Comments in the last column stand for both lines, -> indicates forward and <- indicates reverse direction in the DNA sequence. Inner primers are used for cloning of the knockout construct (TA cloning) and for detection of insertion after *Phormidium* transformation. Outer primers are complementary to sequences outside the knockout construct and are located close to the inner primers on the DNA sequence.

pilA1		
PilA_Fwd	caactacttccgcgctcag	*pilA1* inner primer ->
PilA_Rev	gcagcagattttccagatggtc	<-
PilA_outer_Fwd	gggacttcaggctctcaaatc	*pilA1* outer primer ->
PilA_outer_2_Rev	gtgaagcgtctgactctagc	<-
PilA_short_Fwd	cacaccgctgaggttcatc	*pilA1* inner primer ->
PilA_short_Rev	cttggtcaccgccttcag	<-
KpnI_PilA_Fwd	caacaaggtacccaagttcatccaacacctg	KanR into *pilA1*->
PilA_PacI_Rev	gtggtgttaattaagtttgaaattcagttttcatggttg	<-
pilB		
PilB_Fwd	caaattcttccccttcaggacg	*pilB* inner primer ->
PilB_Rev	cagttgccagtttagtcctgg	<-
PilB_outer_Fwd	gttctccgatggtgtagcc	*pilB* outer primer->
PilB_outer_2_Rev	ggactccatcacatcttcaatcatg	<-
KpnI_PilB_Fwd	caacaaggtaccgtgccttggtggttcag	KanR into *pilB* ->
PilB_PacI_Rev	gtggtgggtacctttgggggcactagaagag	<-
pilD		
PilD_Fwd	ctctttagctattcactaaatagcgc	*pilD* inner primer->
PilD_Rev	cttggatttaaaccgcgct	<-
PilD_outerPCR_Fwd	ctgatgaggtcgccaatc	*pilD* outer primer ->
PilD_outerPCR_Rev	gtggcagattgggatctg	<-
KpnI_PilD_Fwd	caacaaggtaccgccggactgtccctactc	KanR into *pilD* ->
PilD_PacI_Rev	gtggtgttaattaataaatcaccacattggcgaaactcc	<-
pilM		
PilM_Fwd	tgaggaaattaatctcgatgttatacatag	*pilM* inner primer ->
PilM_Rev	gagttaagacctcgcgg	<-
PilM_outerPCR_Fwd	ctcttaaacgattctcttctcgc	*pilM* outer primer ->
PilM_outerPCR_Rev	gtcgttgacattggactgag	<-
KpnI_PilM_Fwd	caacaaggtaccggaagttgaagttaaccactgtatttag	KanR into *pilM* ->
PilM_PacI_Rev	gtggtgttaattaactccaaagccaagaatggc	<-
**pilN**		
PilN_inner_FWD	ctaaattacccctctgcgttg	*pilN* inner primer->
PILN_inner_REV	cactatgtataacatcgagattaatttcc	<-
PilN_fwd_XbaI	caacaatctagacatcaccaccgagggtg	KanR into *pilN* ->
PilN_rev_KpnI	caacaaggtaccattcgcgggatagctcagc	<-
PilN_outer_FWD	gttgtcgtcgtatgccatg	*pilN* outer primer ->
PilN_outer_REV	gactacgggaggtctaacac	<-
***pilQ* (KpnI PacI)**		
PilQ_FWD	ggactcagtggactctg	*pilQ* inner primer ->

PilQ_REV	gggaacgtgaagttacg	<-
SS_KpnI_PilQ_FWD	caacaaggtaccgactcaactgctaacccagc	KanR into *pilQ* ->
SS_PilQ_PacI_REV	gtggtgttaattaagccatctctaccttgcgg	<-
PilQ_outer_FWD	gacgaggaccgacaagga	*pilQ* outer primer ->
PilQ_outer_REV	ccataattggtcgttgatactggtgg	<-
***pilT* (KpnI PacI)**		
PilT_FWD	ccttggaaaaagtgcggg	*pilT*, inner primer ->
PilT_REV	gctctcactttgccctg	<-
SS_KpnI_PilT_FWD	caacaaggtaccttgattgaacaaggtggctc	KanR into *pilT* ->
SS_PilT_PacI_REV	gtggtgttaattaaggactccatcacatcttcaatc	<-
PilT_outer_FWD	gggttggataaactcatttctgatccc	*pilT* outer primer ->
PilT_outer_REV	ccttggctcatcacccc	<-
cphA		
Phyt-Fwd	ccatatcctccagcttagcc	*cphA* inner primer ->
Phyt-rev	gtgacgttccacaatccg	<-
Phyt_KpnI	caacaaggtacccgcaaaatcgcagacc	KanR into *cphA* ->
Phyt_XbaI	caacaatctagaaacccccctctcagttacac	<-
PCR_Phyt fwd	cctcacggaatggtggtgg	*cphA* outer primer ->
PCR_Phyt rev	gccaagagacgattctaccc	<-
**KanR**		
KanR_KpnI	caacaaggtacctaggcaccccaggctttac	diverse primers for KanR cloning
KanR_XbaI	caacaatctagatttgctttgccacggaacg	
PacI_KanRFwd	caacaattaattaataggcaccccaggctttac	
KanR_KpnI_Rev	gtggtgggtacctttgctttgccacggaacg	
KanR_Fwd	ggctcgtatgttgtgtgg	
KanR_Rev	Cgggaagatgcgtgatc	
KanR_Lig_Fwd	Gatcacgcatcttcccg	
KanR_Lig_Rev	Gatccggggaattcgtaatc	
